# Effectiveness of an online educational video intervention to improve the knowledge and behavior of contact lens care during the COVID-19 pandemic: A pre-test/post-test design

**DOI:** 10.1016/j.heliyon.2022.e11009

**Published:** 2022-10-11

**Authors:** Jakkrit Juhong, Auemphon Mordmuang, Juntamanee Jewboonchu, Lunla Udomwech

**Affiliations:** a School of Medicine, Walailak University, Thailand; b Department of Ophthalmology, Walailak University Hospital, Walailak University, Thailand

**Keywords:** Educational video, Health education, Eye care behaviors, Contact lenses, Contact lens hygiene, Contact lens complication

## Abstract

**Objective:**

To assess the effectiveness of an online educational video in improving contact lens (CL) care knowledge and behavior.

**Methods:**

Participants completed a 47-item questionnaire on their CL hygiene knowledge and wear and care behavior. A 5-min CL educational video was shown, and participants completed a post-test. After 2 months, the same questionnaire was used to determine knowledge retention and behavioral changes. Descriptive statistics and McNemar's tests were performed.

**Results:**

The mean age of the 132 enrolled participants was 24 years, and 61% were female. The knowledge scores significantly improved after watching the educational video (p < 0.001). Two months after the intervention, the participants reported changes in their hygienic behavior (p < 0.001).

**Conclusions:**

Online video-based learning is an effective educational tool for improving the knowledge and behavior of CL care.

**Practice implications:**

Patient education via online videos is an innovative and successful strategy that raises awareness, increases patient knowledge, and encourages preventative health behavior to avoid CL-related complications.

## 1 Introduction

Poor knowledge of proper contact lens (CL) hygiene and improper CL care are universal problems, including Thailand [1]. Improper wear and care of CLs can cause a wide range of eye complications from mild conjunctivitis to sight-threatening microbial keratitis [2]. One study reported that 6% of CL users experienced CL-related eye complications each year [3], and the number of those affected continuously rose along with the number of CL wearers. In our previous study [4], we found that nearly 40% of CL wearers had poor CL care compliance. The three most common mistakes were not using CL care solution to clean the storage case, not rubbing and rinsing the lens before storage, and exposing CLs to water during wear. Educational programs on CL care should be emphasized to reduce CL-related eye complications.

As good CL care is the most effective means of preventing CL-related eye infections, educating CL wearers is critical; however, is often not the case. A study reported up to one-third of CL wearers never received instructions for CL use [5, 6]. The current COVID-19 pandemic, lockdown, and social distancing, to all fields of life has been forced to go online, including the medical field as well as patient education. Utilizing online video education eliminated the complexity of scheduling appointment times and travel restrictions. Furthermore, patients could choose a time that suited them with little time burden on the provider and no risk of COVID-19 transmission. Several studies have found that video intervention is a promising educational tool to improve knowledge compared to pamphlets and other methods [7, 8, 9, 10, 11, 12]. However, this patient education method occurred in such different circumstances and lacked real-time response from the educator. In this study, we assessed the effectiveness of an online video intervention on improving knowledge and changing behaviors 2 months afterwards. The results provided evidence supporting the feasibility of an online educational video and its efficacy on changing CL wearers’ behaviors, which could be integrated into public health prevention programs to prevent CL-related complications.

## 2 Methods

This study was conducted between August 2021 and March 2022. Recruitment notices were placed at the Walailak University Hospital's CL Clinic, at various institutions in Thailand, and online. Participants provided informed consent and completed online questionnaires using the Google Forms platform. For this pre-test/post-test study, we estimated that a sample size of 126 participants would be needed to observe a significant difference at an alpha level of 0.05 and 80% power. Participants were eligible if they used CLs at least once weekly in the previous month. This study had no age restrictions; however, participants under the age of 18 gave consent as well as obtained parental or guardian permission before participating. Exclusion criteria included those who could not provide informed consent and those who did not complete the baseline questionnaire. The study protocol followed the tenets of the Declaration of Helsinki and was approved by the Ethics Committee of the Walailak University's Institutional Review Board (WUEC-21-264-01).

Participants completed a pre-test which was a 47 item online questionnaire. The questionnaire was adapted from a previous Thai CL survey [1, 5]. Additional questions were added and modified based on CL care guidelines from the American Academy of Ophthalmology [13]. The questionnaire consisted of three parts. The first part included 19 items about demographic data; the second contained 13 items about the participants' knowledge of CL care, while the last consisted of 15 items about the participants’ everyday behaviors (see the Appendix for the questionnaire). The cumulative session duration was approximately 15 min. The participants watched a 5 min 26 s video after completing the pre-test. It focused on CL wear, handling, and care based on recommendations by the American Academy of Ophthalmology [13]. Participants then completed a post-test after which they were free to watch the movie as often as desired.

### 2.1 Assessment of the CLs used in the participants

The details of the participants’ CLs, such as the type of lens material, replacement plan, frequency of wear in one week, cleaning solution, and source of information on CL handling, were assessed before the video intervention.

### 2.2 Assessment of CL wear and care behavior

CL wear and care behaviors were assessed before and after the video intervention using standard questions from the Thailand CL survey [1, 5] and the American Academy of Ophthalmology on CL care guidelines [13].

### 2.3 Assessment of CL wear and care information retention

Baseline knowledge regarding proper CL wear and care was assessed with a pre-study questionnaire. Retention of the information in the video was assessed immediately and 2 months after watching the video with a post-study questionnaire consisting of the same questions.

### 2.4 Study outcome and statistical analysis

The primary outcome of the study was the participants' knowledge of and behavior regarding CL care. We used descriptive statistics to analyze demographic data and presented the results as mean ± standard deviation (SD), median, frequency, and percentage as appropriate. Mean differences from pre-test to post-test were calculated for knowledge and behavior scores and compared using the McNemar's tests. IBM SPSS Statistics for Windows (version 23.0; SPSS, Chicago, IL, USA) was used to conduct the analyses. Statistical significance was defined as a P value less than 0.05.

## 3 Results

**Figure 1:**
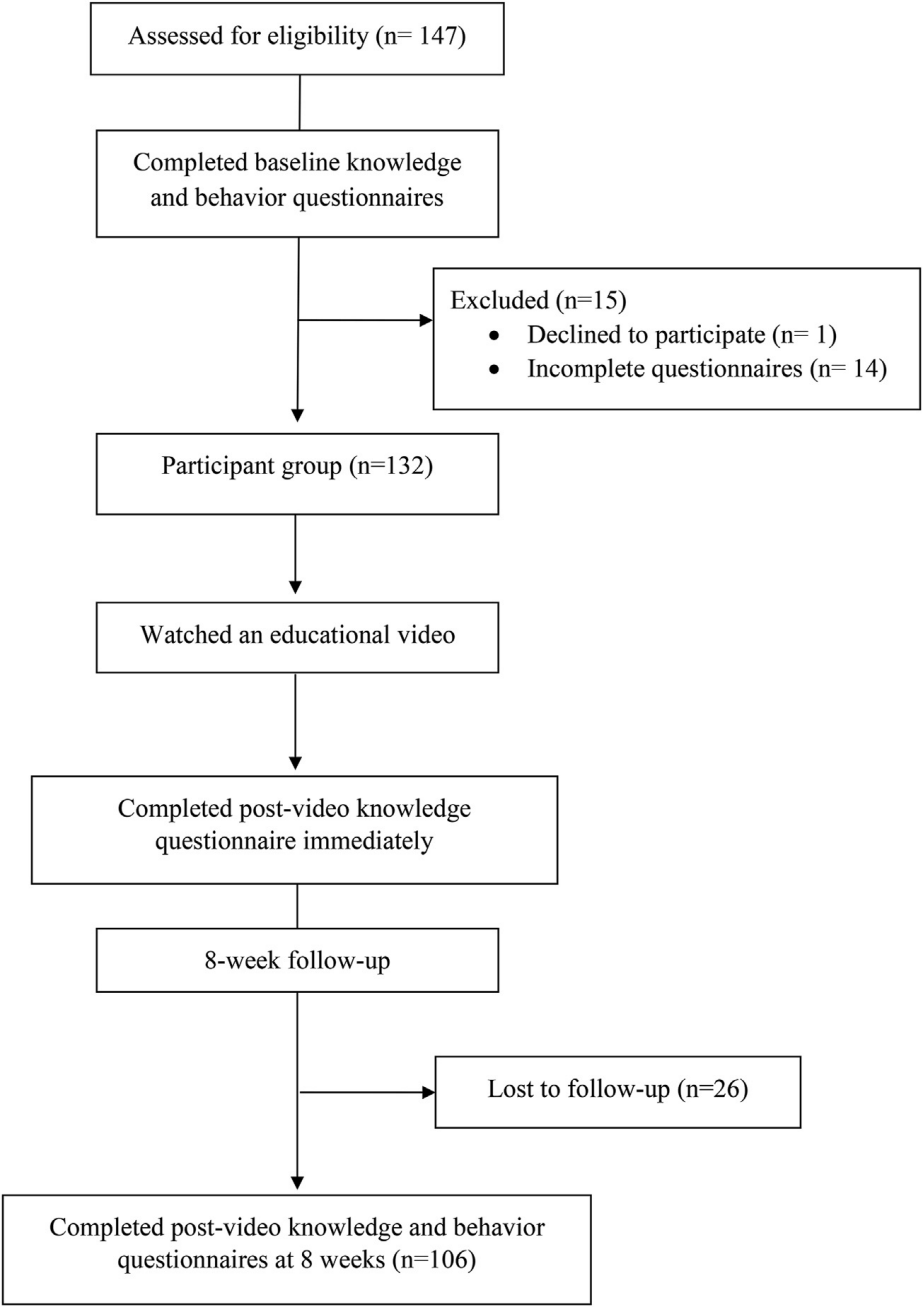
Participant flow diagram.

Of the 147 participants, 15 were excluded because of incomplete answers to the questionnaire. In total, 132 participants completed the study (Figure 1).

### 3.1 Participant demographics and CL characteristics and usage

The general characteristics of the participants are presented in Table 1. Of all the participants, 81 (61.36%) were female, 48 (36.36%) were male, and three (2.27%) identified as LGBTQ+. The average age was 23.41 ± 8.74 years. The median age was 28 years (range: 12–56 years). The education level of 84.84% of participants was high school level or lower. CLs were used mainly to correct refractive errors or for cosmetic purposes, and less than 1% were used for the treatment of corneal disease. Most participants (46.21%) had worn CLs for > a year, 32.58% for 1–5 years, and 21.21% for >5 years. More than 90% wore soft CLs, and 9% wore rigid gas-permeable (RGP) CLs. The popularity of clear, soft CLs was 61.98% and that of cosmetic colored CLs was 38.02%. The majority of participants used CLs every day. The most popular replacement intervals were daily and monthly, which were used by 51.52% and 43.18% of the participants, respectively. More than 50% of participants wore CLs >8 h per day, and >90% purchased CLs and lens care solutions from places without healthcare professionals. Participants mostly consulted opticians or ophthalmologists about starting CL wear; however, > 45% of the participants independently gathered information or received information from friends without recommendations from eye care professionals. The sources of lens care and hygiene instructions were leaflets (56.82%) and verbal advice (18.94%). More than 80% of the participants had experienced CL-related eye symptoms. The top three symptoms recorded in this study were watering, dryness, and irritation. During CL wear, most participants had removed the lens and used eye drops to manage eye discomfort. A minority had eye infections such as keratitis and conjunctivitis associated with CL wear. Only 34% of the participants completed annual eye exams with an ophthalmologist.

**Table 1:** General information of participants.

General information	n = 132 (%)
Sex	
Female	81 (61.36)
Male	48 (36.36)
LGBTQ**+**	3 (2.27)
Age (years old)	
**≤**18	42 (31.82)
19-30	66 (50.00)
31-40	17 (12.88)
41-50	5 (3.79)
**≥**50	2 (1.52)
Underlying disease	
Yes	4 (3.03)
No	128 (96.97)
Education level	
High school or less	56 (42.42)
Vocational/High vocational certificate	5 (3.80)
Graduate	56 (42.42)
Postgraduate	15 (11.36)
Objective	
Refractive errors	61 (46.21)
Refractive errors with cosmetic	50 (37.88)
Cosmetic purposes	19 (14.39)
Treatment of corneal disease	1 (0.76)
Exercise	1 (0.76)
Replacement plan	
Daily	68 (51.52)
Biweekly	7 (5.30)
Monthly	57 (43.18)
Lens wear experience	
Less than 1 years	61 (46.21)
1–5 years	43 (32.58)
6–10 years	16 (12.12)
More than 10 years	12 (9.09)
Type of lens material	
Rigid gas-permeable (RGP) lens	11 (8.33)
Soft CLs	121 (91.67)
- Clear soft CLs	75 (61.98)
- Cosmetic CLs	46 (38.02)
Frequency of wear in a week	
1–3 days	67 (18.66)
4–6 days	33 (32.75)
Everyday	32 (48.59)
Duration of wear	
Less than 8 h	56 (42.42)
More than 8 h	76 (57.58)
Source of CL purchase	
Health care professionals	8 (6.06)
Non-health care professionals	124 (93.94)
Source of disinfecting solution purchase	
Health care professionals	2 (1.52)
Non-health care professionals	130 (98.48)
Whom did you consult when you first started using CLs?	
Ophthalmologist	30 (22.73)
Optometrist	5 (3.79)
Optician	37 (28.03)
Pharmacist	2 (1.52)
Friends	21 (15.91)
None	37 (28.03)
Who explained to you how to put on/remove the lenses and lens care and hygiene?	
Ophthalmologist	29 (21.97)
Optometrist	5 (3.79)
Optician	25 (18.94)
Pharmacist	5 (3.79)
Friends	19 (14.39)
None	48 (36.36)
Did you receive any instructions about lens care and hygiene?	
No	32 (24.24)
Leaflet	75 (56.82)
Oral	25 (18.94)
Symptoms associated with CL wear	
Dryness	28 (21.07)
Irritation	19 (14.05)
Tearing	31 (23.14)
Redness	9 (6.61)
Itchiness	14 (10.74)
Blurry vision	4 (2.90)
Discharge	9 (7.03)
Other	1 (0.41)
None	19 (14.05)
What was your management when you experienced eye discomfort during CL wear?	
Consultation	5 (3.79)
Removing the lenses	55 (41.67)
Self-treatment with antibiotics	3 (2.27)
Use of eye drops	56 (42.42)
No treatment	13 (9.85)
History of eye infection associated with CL wear	
Yes	13 (9.85)
Keratitis	1 (0.76)
Conjunctivitis	12 (9.09)
None	119 (90.15)
Annual eye check-up with ophthalmologist	
No	87 (65.91)
Yes	45 (34.09)

### 3.2 Knowledge improvement

The pre-test consisted of true-false questions to determine the participants' understanding of CL wear and care behaviors. The results showed that > half of the participants misunderstood proper CL hygiene. They mistook correct CL hygiene as cleaning the CL with normal saline (60.45%), not changing lens care solutions after 3 months (94.78%), not changing storage cases after 3 months (97.76%), not cleaning storage cases with lens care solution (67.16%), and not cleaning the lens cases daily (69.40%). The improvement in the participants’ knowledge was evaluated by an immediate post-test and a 2-month final test. The answers to each question were compared with prior results. The number of correct answers to each question was represented as a percentage of the participants (Table 2). Immediately and 2 months after watching the video, participants answered more questions correctly. The increase in correctly answers was statistically significant for all questions. The questions that received 100% correct responses in the final test included items regarding the use of drinking water, normal saline, and saliva to clean CLs; closing the cap of the cleaning solution tightly after use; using cleaning solution for cleaning the lens cases; and cleaning the case daily after usage.

**Table 2:** CL care knowledge before (pre-test), immediately after (post-test), and 2 months after (final test) watching an educational video.

Knowledge questions	Correct answer	Percentage of participants with correct answers				
		Pre-test (n = 132)	Immediate		After 2 months	
			Post-test (n = 132)	p-value	Final test (n = 106)	p-value
CLs can be cleaned with drinking water	False	76.12	92.54	<0.001	100.00	<0.001
CLs can be cleaned with normal saline	False	39.55	93.28	<0.001	100.00	<0.001
CLs can be cleaned with saliva	False	75.37	98.51	<0.001	100.00	<0.001
Dropped CLs can be used after cleaning	True	61.19	98.51	<0.001	99.06	<0.001
One should start inserting and removing the lens from the same eye	True	75.37	97.01	<0.001	94.34	<0.001
CLs should be soaked in cleaning solution at least 6 h before reuse	True	85.82	98.51	<0.001	95.28	<0.001
The CL case should be filled with fresh CL solution everyday	True	94.78	94.78	1.000	99.06	1.000
Topping off the old cleaning solution in the CL case is acceptable	False	72.39	97.76	<0.001	99.06	<0.001
The cap of the cleaning solution should be tightly closed after use	True	79.10	92.54	0.001	100.00	0.001
How often should the CL solutions be changed?	3 months	5.22	83.58	<0.001	94.34	<0.001
How often should the CL case be changed?	3 months	2.24	81.34	<0.001	85.85	<0.001
What should be used to clean the CL cases?	CL cleaning solution	32.84	90.30	<0.001	100.00	<0.001
How often should the CL case be cleaned?	everyday	30.60	84.33	<0.001	100.00	<0.001

### 3.3 Improvement in CL wear and care behaviors

The participants' CL wear and care behaviors were examined before watching the video and 2 months after. Responses to each question were reported as the percentage of participants that answered correctly (Table 3). At baseline, improper behaviors included water exposure such as swimming (37.12%), washing the face (36.36%), and taking showers (27.27%) while wearing CLs. Other risky behaviors included sleeping with CLs (15.15%), sharing CLs with others (7.58%), exceeding the manufacturers' recommended replacement period (15.91%), and using expired CL solutions (13.64%). In the pre-test, > 60% of participants reported good CL wear and care practices such as checking the expiration date and integrity of packaging before use, checking for the correct CL side before insertion, washing hands with soap and drying before CL handling, and rubbing and rinsing CLs after removal. Behaviors improved 2 months after the video intervention. The prior observed risky practices decreased among participants and no participants shared CLs with others, exceeded the manufacturers’ recommended planned replacement period, used expired CL solutions, or wore CLs in the shower. Moreover, good CL wear and care behaviors were completely correct in all participants at the 2 month period.

**Table 3:** CL wear and care behaviors.

Behavior of CL wear	Pre-test n = 132 (%)	Post-test after 2 months n = 106 (%)	p-value
Sleeping with CLs	20 (15.15)	5 (4.72)	0.007
Sharing CLs with others	10 (7.58)	0 (0.00)	0.004
Exceeding the CL's recommended replacement period	21 (15.91)	0 (0.00)	<0.001
Using expired CL solutions (opened for more than 3 months)	18 (13.64)	0 (0.00)	<0.001
Swimming while wearing CLs	49 (37.12)	5 (4.72)	<0.001
Washing your face while wearing CLs	48 (36.36)	3 (2.83)	<0.001
Showering while wearing CLs	36 (27.27)	0 (0.00)	<0.001
Always checking the expiration date and integrity of packaging before use	120 (90.91)	106 (100.00)	0.004
Checking for the correct CL side (inside-outside) before use	123 (93.18)	106 (100.00)	0.031
Hand washing with soap before inserting the CLs	94 (71.21)	102 (100.00)	0.021
Drying hands before inserting CLs with a lint-free ​towel	88 (66.67)	105 (100.00)	<0.001
Rubbing and rinsing CLs before inserting the CLs	53 (40.15)	106 (100.00)	<0.001
Hand washing with soap before CL removal	95 (71.97)	106 (100.00)	0.016
Drying hands before CL removal with a lint-free ​towel	96 (72.73)	106 (100.00)	<0.001
Rubbing and rinsing CLs after CL removal	80 (60.61)	106 (100.00)	<0.001

## 4 Discussion and conclusion

### 4.1 Discussion

Approximately 125 million people (2% of the population) use CLs worldwide [14]. Consistent with our findings, most CL wearers globally are female and relatively young [14]. In Thailand, wearing CLs is popular among high school, college, and university students [15], many of whom may be at a higher risk of complications because of improper wear and care behaviors [16]. According to a previous study [17], >99% of CL wearers engaged in unsanitary practices that exposed them to eye infections and other CL-related complications. The most common unfavorable behaviors were failing to use CL care solution to clean the storage case, failing to rub and wash CLs before storage, exposing CLs to water while wearing, using makeup around the ocular area, and missing annual eye examinations [3]. The reasons for inadequate CL care are varied and complex [18]. Inadequate knowledge of CL wear and care, of proper hygiene practices, and of CL complications contribute to this problem [19]. This knowledge is important and necessary as health education on CL care behaviors helps prevent CL-related complications [15]; however, correct information is not always taught by healthcare providers or ophthalmologist. In the COVID-19 pandemic era, education has moved towards online teaching. To address these issues, our online educational video aimed to increase knowledge regarding CL use and promote behavioral changes in CL handling. Several meta-analyses have found that brief educational videos can improve learning and short-term knowledge [7, 8], and multiple studies have found that videos can be as effective as or better than traditional patient education materials [9, 10, 11, 12]. A randomized, controlled trial by Armstrong et al. [20] reported that video intervention is a promising educational method to improve knowledge compared to pamphlets, as well as to promote a change in the behavior to a more proper use of medicinal products. Even short educational videos can influence individuals to progress through stages of change to reach their desired health goals [21]. One study found that readiness to change significantly increased after watching videos, suggesting that watching videos helped these individuals move closer to actively improving their objective outcomes [22]. A study on end-of-life care found that video education had a greater effect than verbal education on patients choosing comfort care over life-prolonging care, which was the goal. This supports the idea that a video can communicate a complicated and multi-dimensional message and facilitate decision making. Our educational video not only increased patients’ knowledge, but also improved CL behavior after 2 months [23]. Interestingly, in this study, high knowledge retention could be attributed to using an online platform that participants could review as needed. This allowed participants to self-practice while emphasizing accurate CL hygiene.

To our knowledge, this is the first study to evaluate the effectiveness of educational videos on CL care knowledge and behavior. The statistical power to detect a significant effect on the primary outcome was more than adequate. The limitations of this study include the use of a video designed for an adult, Thai population; thus it might not be generalizable to other ages and countries. Furthermore because the video was online, we were unable to determine the level of engagement or whether the participants viewed the entire video or checked out the video every time when they are using the CL; however, the increase in post-test knowledge and improved behavior within 2 months suggests adequate participant engagement. Finally, because there was no control (face-to-face education) group in this study, the external validity was limited; however, the immediacy of the post-test should help counteract this. Further real-world studies are required to address the effects of educational videos.

## 5 Conclusions

Patient education is a critical component of primary preventive approaches for CL-related complications caused by improper wear and care. In this study, we evaluated the effectiveness of online videos as an educational strategy for CL wearers. Participants who watched an online educational video scored higher on a questionnaire immediately after and 2 months after the video than on the questionnaire before watching the video. Furthermore, their CL hygiene practices improved 2 months after watching the video.

### 5.1 Practice implications

These findings support the use of online videos to educate patients about appropriate CL wear and care practices. Online video intervention is free and may be conducted in any eye care clinic during routine care without consuming excessive time. Patients can easily access these videos on YouTube or through an institution's web resources. Patient education via online videos may become an innovative and successful technique to increase patient knowledge and long-term health behaviors.

## Declarations

### Author contribution statement

Jakkrit Juhong, M. D: Conceived and designed the experiments; Performed the experiments; Analyzed and interpreted the data; Contributed reagents, materials, analysis tools or data; Wrote the paper.

Lunla Udomwech: Performed the experiments; Wrote the paper.

Auemphon Mordmuang: Analyzed and interpreted the data; Wrote the paper.

Juntamanee Jewboonchu: Performed the experiments; Contributed reagents, materials, analysis tools or data.

### Funding statement

Dr. Jakkrit Juhong was supported by 10.13039/501100021365Institute of Research and Innovation, Walailak University [WU-IRG-64-071, 2021].

### Data availability statement

Data included in article/supp. material/referenced in article.

### Declaration of interest's statement

The authors declare no conflict of interest.

### Additional information

No additional information is available for this paper.

## Appendix A Supplementary data

Behavior Subgroup Analysis Behavior Subgroup Analysis

Knowledge Subgroup Analysis Knowledge Subgroup Analysis
