# Supplementary material for: Effectiveness of an online educational video intervention to improve the knowledge and behavior of contact lens care during the COVID-19 pandemic: A pre-test/post-test design
Source: Heliyon. 2022 Oct 11;8(10):e11009. doi: 10.1016/j.heliyon.2022.e11009 (PMC9551115; doi:10.1016/j.heliyon.2022.e11009)
Supplement: Behavior Subgroup Analysis [file mmc1.pdf]

Your license will expire in 22 days.

Crosstabs

Notes

Output Created		09-SEP-2022 16:22:11
Comments		
Input	Data	/Users/Jacky/Desktop/SPSS CL modality/PrePostTest_Behavior.sav
	Active Dataset	DataSet1
	Filter	<none>
	Weight	<none>
	Split File	Schedule
	N of Rows in Working Data File	132
Missing Value Handling	Definition of Missing	User-defined missing values are treated as missing.
	Cases Used	Statistics for each table are based on all the cases with valid data in the specified range(s) for all variables in each table.
Syntax		CROSSTABS /TABLES=Pretest1 BY Posttest1 /FORMAT=AVALUE TABLES /STATISTICS=MCNEMAR /CELLS=COUNT TOTAL /COUNT ROUND CELL.
Resources	Processor Time	00:00:00.03
	Elapsed Time	00:00:00.00
	Dimensions Requested	2
	Cells Available	524245

Warnings

No measures of association are computed for the crosstabulation of Pretest 1 * Post test 1 for split file Schedule=RGP permanent. At least one variable in each 2-way table upon which measures of association are computed is a constant.
No measures of association are computed for the crosstabulation of Pretest 1 * Post test 1 for split file Schedule=Soft CL Biweekly. At least one variable in each 2-way table upon which measures of association are computed is a constant.
No measures of association are computed for the crosstabulation of Pretest 1 * Post test 1 for split file Schedule=Soft CL Monthly. At least one variable in each 2-way table upon which measures of association are computed is a constant.

Case Processing Summary

		Cases			
		Valid		Missing	
Schedule		N	Percent	N	Percent
RGP permanent	Pretest 1 * Post test 1	6	60.0%	4	40.0%
Soft CL Daily	Pretest 1 * Post test 1	49	83.1%	10	16.9%
Soft CL Biweekly	Pretest 1 * Post test 1	6	85.7%	1	14.3%
Soft CL Monthly	Pretest 1 * Post test 1	54	96.4%	2	3.6%

Case Processing Summary

		Cases	
		Total	
Schedule		N	Percent
RGP permanent	Pretest 1 * Post test 1	10	100.0%
Soft CL Daily	Pretest 1 * Post test 1	59	100.0%
Soft CL Biweekly	Pretest 1 * Post test 1	7	100.0%
Soft CL Monthly	Pretest 1 * Post test 1	56	100.0%

Pretest 1 * Post test 1 Crosstabulation

Schedule			Post test 1		Total
RGP permanent	Pretest 1	Count		6	6
		% of Total		100.0%	100.0%
	Total	Count		6	6
		% of Total		100.0%	100.0%
Soft CL Daily	Pretest 1	Count	0	3	3
		% of Total	0.0%	6.1%	6.1%
		Count	5	41	46
		% of Total	10.2%	83.7%	93.9%
	Total	Count	5	44	49
		% of Total	10.2%	89.8%	100.0%
Soft CL Biweekly	Pretest 1	Count		6	6
		% of Total		100.0%	100.0%
	Total	Count		6	6
		% of Total		100.0%	100.0%
Soft CL Monthly	Pretest 1	Count		16	16
		% of Total		29.6%	29.6%
		Count		38	38
		% of Total		70.4%	70.4%
	Total	Count		54	54
		% of Total		100.0%	100.0%

Chi-Square Tests

Schedule		Value	df	Asymptotic Significance (2-sided)	Exact Sig. (2-sided)
RGP permanent	McNemar-Bowker Test	.	.	.[a]	
	N of Valid Cases	6			
Soft CL Daily	N of Valid Cases	49			
	McNemar Test				.727[b]
Soft CL Biweekly	McNemar-Bowker Test	.	.	.[a]	
	N of Valid Cases	6			
Soft CL Monthly	McNemar-Bowker Test	.	.	.[a]	
	N of Valid Cases	54			

a. Computed only for a PxP table, where P must be greater than 1.

b. Binomial distribution used.

Crosstabs

Notes

Output Created		09-SEP-2022 16:23:34
Comments		
Input	Data	/Users/Jacky/Desktop/SPSS CL modality/PrePostTest_Behavior.sav
	Active Dataset	DataSet1
	Filter	<none>
	Weight	<none>
	Split File	Schedule
	N of Rows in Working Data File	132
Missing Value Handling	Definition of Missing	User-defined missing values are treated as missing.
	Cases Used	Statistics for each table are based on all the cases with valid data in the specified range(s) for all variables in each table.
Syntax		CROSSTABS /TABLES=Pretest2 BY Posttest2 /FORMAT=AVALUE TABLES /STATISTICS=MCNEMAR /CELLS=COUNT TOTAL /COUNT ROUND CELL.
Resources	Processor Time	00:00:00.02
	Elapsed Time	00:00:00.00
	Dimensions Requested	2
	Cells Available	524245

Warnings

No measures of association are computed for the crosstabulation of Pretest 2 * Post test 2 for split file Schedule=RGP permanent. At least one variable in each 2-way table upon which measures of association are computed is a constant.
No measures of association are computed for the crosstabulation of Pretest 2 * Post test 2 for split file Schedule=Soft CL Daily. At least one variable in each 2-way table upon which measures of association are computed is a constant.
No measures of association are computed for the crosstabulation of Pretest 2 * Post test 2 for split file Schedule=Soft CL Biweekly. At least one variable in each 2-way table upon which measures of association are computed is a constant.
No measures of association are computed for the crosstabulation of Pretest 2 * Post test 2 for split file Schedule=Soft CL Monthly. At least one variable in each 2-way table upon which measures of association are computed is a constant.

Case Processing Summary

		Cases			
		Valid		Missing	
Schedule		N	Percent	N	Percent
RGP permanent	Pretest 2 * Post test 2	5	50.0%	5	50.0%
Soft CL Daily	Pretest 2 * Post test 2	47	79.7%	12	20.3%
Soft CL Biweekly	Pretest 2 * Post test 2	5	71.4%	2	28.6%
Soft CL Monthly	Pretest 2 * Post test 2	49	87.5%	7	12.5%

Case Processing Summary

		Cases	
		Total	
Schedule		N	Percent
RGP permanent	Pretest 2 * Post test 2	10	100.0%
Soft CL Daily	Pretest 2 * Post test 2	59	100.0%
Soft CL Biweekly	Pretest 2 * Post test 2	7	100.0%
Soft CL Monthly	Pretest 2 * Post test 2	56	100.0%

Pretest 2 * Post test 2 Crosstabulation

Schedule			Post test 2	Total
RGP permanent	Pretest 2	Count	5	5
		% of Total	100.0%	100.0%
	Total	Count	5	5
		% of Total	100.0%	100.0%
Soft CL Daily	Pretest 2	Count	3	3
		% of Total	6.4%	6.4%
		Count	44	44
		% of Total	93.6%	93.6%
	Total	Count	47	47
		% of Total	100.0%	100.0%
Soft CL Biweekly	Pretest 2	Count	5	5
		% of Total	100.0%	100.0%
	Total	Count	5	5
		% of Total	100.0%	100.0%
Soft CL Monthly	Pretest 2	Count	6	6
		% of Total	12.2%	12.2%
		Count	43	43
		% of Total	87.8%	87.8%
	Total	Count	49	49
		% of Total	100.0%	100.0%

Chi-Square Tests

Schedule		Value	df	Asymptotic Significance (2-sided)
RGP permanent	McNemar-Bowker Test	.	.	.[a]
	N of Valid Cases	5		
Soft CL Daily	McNemar-Bowker Test	.	.	.[a]
	N of Valid Cases	47		
Soft CL Biweekly	McNemar-Bowker Test	.	.	.[a]
	N of Valid Cases	5		
Soft CL Monthly	McNemar-Bowker Test	.	.	.[a]
	N of Valid Cases	49		

a. Computed only for a PxP table, where P must be greater than 1.

Crosstabs

Notes

Output Created		09-SEP-2022 16:24:42
Comments		
Input	Data	/Users/Jacky/Desktop/SPSS CL modality/PrePostTest_Behavior.sav
	Active Dataset	DataSet1
	Filter	<none>
	Weight	<none>
	Split File	Schedule
	N of Rows in Working Data File	132
Missing Value Handling	Definition of Missing	User-defined missing values are treated as missing.
	Cases Used	Statistics for each table are based on all the cases with valid data in the specified range(s) for all variables in each table.
Syntax		CROSSTABS<br>/TABLES=Pretest3 BY Posttest3<br>/FORMAT=AVALUE TABLES<br>/STATISTICS=MCNEMAR<br>/CELLS=COUNT TOTAL<br>/COUNT ROUND CELL.
Resources	Processor Time	00:00:00.03
	Elapsed Time	00:00:00.00
	Dimensions Requested	2
	Cells Available	524245

Warnings

No measures of association are computed for the crosstabulation of Pretest 3 * Post test 3 for split file Schedule=RGP permanent. At least one variable in each 2-way table upon which measures of association are computed is a constant.
No measures of association are computed for the crosstabulation of Pretest 3 * Post test 3 for split file Schedule=Soft CL Daily. At least one variable in each 2-way table upon which measures of association are computed is a constant.
No measures of association are computed for the crosstabulation of Pretest 3 * Post test 3 for split file Schedule=Soft CL Biweekly. At least one variable in each 2-way table upon which measures of association are computed is a constant.
No measures of association are computed for the crosstabulation of Pretest 3 * Post test 3 for split file Schedule=Soft CL Monthly. At least one variable in each 2-way table upon which measures of association are computed is a constant.

Case Processing Summary

		Cases			
		Valid		Missing	
Schedule		N	Percent	N	Percent
RGP permanent	Pretest 3 * Post test 3	5	50.0%	5	50.0%
Soft CL Daily	Pretest 3 * Post test 3	47	79.7%	12	20.3%
Soft CL Biweekly	Pretest 3 * Post test 3	5	71.4%	2	28.6%
Soft CL Monthly	Pretest 3 * Post test 3	49	87.5%	7	12.5%

Case Processing Summary

		Cases	
		Total	
Schedule		N	Percent
RGP permanent	Pretest 3 * Post test 3	10	100.0%
Soft CL Daily	Pretest 3 * Post test 3	59	100.0%
Soft CL Biweekly	Pretest 3 * Post test 3	7	100.0%
Soft CL Monthly	Pretest 3 * Post test 3	56	100.0%

Pretest 3 * Post test 3 Crosstabulation

Schedule			Post test 3	Total
RGP permanent	Pretest 3	Count	1	1
		% of Total	20.0%	20.0%
		Count	4	4
		% of Total	80.0%	80.0%
	Total	Count	5	5
		% of Total	100.0%	100.0%
Soft CL Daily	Pretest 3	Count	7	7
		% of Total	14.9%	14.9%
		Count	40	40
		% of Total	85.1%	85.1%
	Total	Count	47	47
		% of Total	100.0%	100.0%
Soft CL Biweekly	Pretest 3	Count	5	5
		% of Total	100.0%	100.0%
	Total	Count	5	5
		% of Total	100.0%	100.0%
Soft CL Monthly	Pretest 3	Count	12	12
		% of Total	24.5%	24.5%
		Count	37	37
		% of Total	75.5%	75.5%
	Total	Count	49	49
		% of Total	100.0%	100.0%

Chi-Square Tests

Schedule		Value	df	Asymptotic Significance (2-sided)
RGP permanent	McNemar-Bowker Test	.	.	.[a]
	N of Valid Cases	5		
Soft CL Daily	McNemar-Bowker Test	.	.	.[a]
	N of Valid Cases	47		
Soft CL Biweekly	McNemar-Bowker Test	.	.	.[a]
	N of Valid Cases	5		
Soft CL Monthly	McNemar-Bowker Test	.	.	.[a]
	N of Valid Cases	49		

a. Computed only for a PxP table, where P must be greater than 1.

Crosstabs

Notes

Output Created		09-SEP-2022 16:25:18
Comments		
Input	Data	/Users/Jacky/Desktop/SPSS CL modality/PrePostTest_Behavior.sav
	Active Dataset	DataSet1
	Filter	<none>
	Weight	<none>
	Split File	Schedule
	N of Rows in Working Data File	132
Missing Value Handling	Definition of Missing	User-defined missing values are treated as missing.
	Cases Used	Statistics for each table are based on all the cases with valid data in the specified range(s) for all variables in each table.
Syntax		CROSSTABS<br>/TABLES=Pretest4 BY Posttest4<br>/FORMAT=AVALUE TABLES<br>/STATISTICS=MCNEMAR<br>/CELLS=COUNT TOTAL<br>/COUNT ROUND CELL.
Resources	Processor Time	00:00:00.02
	Elapsed Time	00:00:00.00
	Dimensions Requested	2
	Cells Available	524245

Warnings

No measures of association are computed for the crosstabulation of Pretest 4 * Post test 4 for split file Schedule=RGP permanent. At least one variable in each 2-way table upon which measures of association are computed is a constant.
No measures of association are computed for the crosstabulation of Pretest 4 * Post test 4 for split file Schedule=Soft CL Daily. At least one variable in each 2-way table upon which measures of association are computed is a constant.
No measures of association are computed for the crosstabulation of Pretest 4 * Post test 4 for split file Schedule=Soft CL Biweekly. At least one variable in each 2-way table upon which measures of association are computed is a constant.
No measures of association are computed for the crosstabulation of Pretest 4 * Post test 4 for split file Schedule=Soft CL Monthly. At least one variable in each 2-way table upon which measures of association are computed is a constant.

Case Processing Summary

		Cases			
		Valid		Missing	
Schedule		N	Percent	N	Percent
RGP permanent	Pretest 4 * Post test 4	5	50.0%	5	50.0%
Soft CL Daily	Pretest 4 * Post test 4	47	79.7%	12	20.3%
Soft CL Biweekly	Pretest 4 * Post test 4	5	71.4%	2	28.6%
Soft CL Monthly	Pretest 4 * Post test 4	49	87.5%	7	12.5%

Case Processing Summary

		Cases	
		Total	
Schedule		N	Percent
RGP permanent	Pretest 4 * Post test 4	10	100.0%
Soft CL Daily	Pretest 4 * Post test 4	59	100.0%
Soft CL Biweekly	Pretest 4 * Post test 4	7	100.0%
Soft CL Monthly	Pretest 4 * Post test 4	56	100.0%

Pretest 4 * Post test 4 Crosstabulation

Schedule			Post test 4	Total
RGP permanent	Pretest 4	Count	5	5
		% of Total	100.0%	100.0%
	Total	Count	5	5
		% of Total	100.0%	100.0%
Soft CL Daily	Pretest 4	Count	6	6
		% of Total	12.8%	12.8%
		Count	41	41
		% of Total	87.2%	87.2%
	Total	Count	47	47
		% of Total	100.0%	100.0%
Soft CL Biweekly	Pretest 4	Count	5	5
		% of Total	100.0%	100.0%
	Total	Count	5	5
		% of Total	100.0%	100.0%
Soft CL Monthly	Pretest 4	Count	11	11
		% of Total	22.4%	22.4%
		Count	38	38
		% of Total	77.6%	77.6%
	Total	Count	49	49
		% of Total	100.0%	100.0%

Chi-Square Tests

Schedule		Value	df	Asymptotic Significance (2-sided)
RGP permanent	McNemar-Bowker Test	.	.	.[a]
	N of Valid Cases	5		
Soft CL Daily	McNemar-Bowker Test	.	.	.[a]
	N of Valid Cases	47		
Soft CL Biweekly	McNemar-Bowker Test	.	.	.[a]
	N of Valid Cases	5		
Soft CL Monthly	McNemar-Bowker Test	.	.	.[a]
	N of Valid Cases	49		

a. Computed only for a PxP table, where P must be greater than 1.

Crosstabs

Notes

Output Created		09-SEP-2022 16:25:28
Comments		
Input	Data	/Users/Jacky/Desktop/SPSS CL modality/PrePostTest_Behavior.sav
	Active Dataset	DataSet1
	Filter	<none>
	Weight	<none>
	Split File	Schedule
	N of Rows in Working Data File	132
Missing Value Handling	Definition of Missing	User-defined missing values are treated as missing.
	Cases Used	Statistics for each table are based on all the cases with valid data in the specified range(s) for all variables in each table.
Syntax		CROSSTABS /TABLES=Pretest5 BY Posttest5 /FORMAT=AVALUE TABLES /STATISTICS=MCNEMAR /CELLS=COUNT TOTAL /COUNT ROUND CELL.
Resources	Processor Time	00:00:00.02
	Elapsed Time	00:00:00.00
	Dimensions Requested	2
	Cells Available	524245

Warnings

No measures of association are computed for the crosstabulation of Pretest 5 * Post test 5 for split file Schedule=RGP permanent. At least one variable in each 2-way table upon which measures of association are computed is a constant.
No measures of association are computed for the crosstabulation of Pretest 5 * Post test 5 for split file Schedule=Soft CL Biweekly. At least one variable in each 2-way table upon which measures of association are computed is a constant.

Case Processing Summary

Schedule		Cases			
		Valid		Missing	
		N	Percent	N	Percent
RGP permanent	Pretest 5 * Post test 5	5	50.0%	5	50.0%
Soft CL Daily	Pretest 5 * Post test 5	47	79.7%	12	20.3%
Soft CL Biweekly	Pretest 5 * Post test 5	5	71.4%	2	28.6%
Soft CL Monthly	Pretest 5 * Post test 5	49	87.5%	7	12.5%

Case Processing Summary

Schedule		Cases	
		Total	
		N	Percent
RGP permanent	Pretest 5 * Post test 5	10	100.0%
Soft CL Daily	Pretest 5 * Post test 5	59	100.0%
Soft CL Biweekly	Pretest 5 * Post test 5	7	100.0%
Soft CL Monthly	Pretest 5 * Post test 5	56	100.0%

Pretest 5 * Post test 5 Crosstabulation

Schedule			Post test 5		Total
RGP permanent	Pretest 5	Count		5	5
		% of Total		100.0%	100.0%
	Total	Count		5	5
		% of Total		100.0%	100.0%
Soft CL Daily	Pretest 5	Count	0	14	14
		% of Total	0.0%	29.8%	29.8%
		Count	4	29	33
		% of Total	8.5%	61.7%	70.2%
	Total	Count	4	43	47
		% of Total	8.5%	91.5%	100.0%
Soft CL Biweekly	Pretest 5	Count		5	5
		% of Total		100.0%	100.0%
	Total	Count		5	5
		% of Total		100.0%	100.0%
Soft CL Monthly	Pretest 5	Count	0	30	30
		% of Total	0.0%	61.2%	61.2%
		Count	1	18	19
		% of Total	2.0%	36.7%	38.8%
	Total	Count	1	48	49
		% of Total	2.0%	98.0%	100.0%

Chi-Square Tests

Schedule		Value	df	Asymptotic Significance (2-sided)	Exact Sig. (2-sided)
RGP permanent	McNemar-Bowker Test	.	.	.[a]	
	N of Valid Cases	5			
Soft CL Daily	N of Valid Cases	47			
	McNemar Test				.031[b]
Soft CL Biweekly	McNemar-Bowker Test	.	.	.[a]	
	N of Valid Cases	5			
Soft CL Monthly	N of Valid Cases	49			
	McNemar Test				<.001[b]

a. Computed only for a PxP table, where P must be greater than 1.

b. Binomial distribution used.

Crosstabs

Notes

Output Created		09-SEP-2022 16:26:04
Comments		
Input	Data	/Users/Jacky/Desktop/SPSS CL modality/PrePostTest_Behavior.sav
	Active Dataset	DataSet1
	Filter	<none>
	Weight	<none>
	Split File	Schedule
	N of Rows in Working Data File	132
Missing Value Handling	Definition of Missing	User-defined missing values are treated as missing.
	Cases Used	Statistics for each table are based on all the cases with valid data in the specified range(s) for all variables in each table.
Syntax		CROSSTABS<br>/TABLES=Pretest9 BY Posttest9<br>/FORMAT=AVALUE TABLES<br>/STATISTICS=MCNEMAR<br>/CELLS=COUNT TOTAL<br>/COUNT ROUND CELL.
Resources	Processor Time	00:00:00.03
	Elapsed Time	00:00:01.00
	Dimensions Requested	2
	Cells Available	524245

Warnings

No measures of association are computed for the crosstabulation of Pretest 9 * Post test 9 for split file Schedule=RGP permanent. At least one variable in each 2-way table upon which measures of association are computed is a constant.
No measures of association are computed for the crosstabulation of Pretest 9 * Post test 9 for split file Schedule=Soft CL Biweekly. At least one variable in each 2-way table upon which measures of association are computed is a constant.

Case Processing Summary

Schedule		Cases			
		Valid		Missing	
		N	Percent	N	Percent
RGP permanent	Pretest 9 * Post test 9	5	50.0%	5	50.0%
Soft CL Daily	Pretest 9 * Post test 9	47	79.7%	12	20.3%
Soft CL Biweekly	Pretest 9 * Post test 9	5	71.4%	2	28.6%
Soft CL Monthly	Pretest 9 * Post test 9	49	87.5%	7	12.5%

Case Processing Summary

Schedule		Cases	
		Total	
		N	Percent
RGP permanent	Pretest 9 * Post test 9	10	100.0%
Soft CL Daily	Pretest 9 * Post test 9	59	100.0%
Soft CL Biweekly	Pretest 9 * Post test 9	7	100.0%
Soft CL Monthly	Pretest 9 * Post test 9	56	100.0%

Pretest 9 * Post test 9 Crosstabulation

Schedule			Post test 9		Total
RGP permanent	Pretest 9	Count		5	5
		% of Total		100.0%	100.0%
	Total	Count		5	5
		% of Total		100.0%	100.0%
Soft CL Daily	Pretest 9	Count	1	6	7
		% of Total	2.1%	12.8%	14.9%
		Count	1	39	40
		% of Total	2.1%	83.0%	85.1%
	Total	Count	2	45	47
		% of Total	4.3%	95.7%	100.0%
Soft CL Biweekly	Pretest 9	Count		1	1
		% of Total		20.0%	20.0%
		Count		4	4
		% of Total		80.0%	80.0%
	Total	Count		5	5
		% of Total		100.0%	100.0%
Soft CL Monthly	Pretest 9	Count	0	34	34
		% of Total	0.0%	69.4%	69.4%
		Count	1	14	15
		% of Total	2.0%	28.6%	30.6%
	Total	Count	1	48	49
		% of Total	2.0%	98.0%	100.0%

Chi-Square Tests

Schedule		Value	df	Asymptotic Significance (2-sided)	Exact Sig. (2-sided)
RGP permanent	McNemar-Bowker Test	.	.	.[a]	
	N of Valid Cases	5			
Soft CL Daily	N of Valid Cases	47			
	McNemar Test				.125[b]
Soft CL Biweekly	McNemar-Bowker Test	.	.	.[a]	
	N of Valid Cases	5			
Soft CL Monthly	N of Valid Cases	49			
	McNemar Test				<.001[b]

a. Computed only for a PxP table, where P must be greater than 1.

b. Binomial distribution used.

Crosstabs

Notes

Output Created		09-SEP-2022 16:26:30
Comments		
Input	Data	/Users/Jacky/Desktop/SPSS CL modality/PrePostTest_Behavior.sav
	Active Dataset	DataSet1
	Filter	<none>
	Weight	<none>
	Split File	Schedule
	N of Rows in Working Data File	132
Missing Value Handling	Definition of Missing	User-defined missing values are treated as missing.
	Cases Used	Statistics for each table are based on all the cases with valid data in the specified range(s) for all variables in each table.
Syntax		CROSSTABS /TABLES=Pretest8 BY Posttest8 /FORMAT=AVALUE TABLES /STATISTICS=MCNEMAR /CELLS=COUNT TOTAL /COUNT ROUND CELL.
Resources	Processor Time	00:00:00.02
	Elapsed Time	00:00:00.00
	Dimensions Requested	2
	Cells Available	524245

Warnings

No measures of association are computed for the crosstabulation of Pretest 8 * Post test 8 for split file Schedule=RGP permanent. At least one variable in each 2-way table upon which measures of association are computed is a constant.
No measures of association are computed for the crosstabulation of Pretest 8 * Post test 8 for split file Schedule=Soft CL Daily. At least one variable in each 2-way table upon which measures of association are computed is a constant.
No measures of association are computed for the crosstabulation of Pretest 8 * Post test 8 for split file Schedule=Soft CL Biweekly. At least one variable in each 2-way table upon which measures of association are computed is a constant.
No measures of association are computed for the crosstabulation of Pretest 8 * Post test 8 for split file Schedule=Soft CL Monthly. At least one variable in each 2-way table upon which measures of association are computed is a constant.

Case Processing Summary

		Cases			
		Valid		Missing	
Schedule		N	Percent	N	Percent
RGP permanent	Pretest 8 * Post test 8	5	50.0%	5	50.0%
Soft CL Daily	Pretest 8 * Post test 8	47	79.7%	12	20.3%
Soft CL Biweekly	Pretest 8 * Post test 8	5	71.4%	2	28.6%
Soft CL Monthly	Pretest 8 * Post test 8	49	87.5%	7	12.5%

Case Processing Summary

		Cases	
		Total	
Schedule		N	Percent
RGP permanent	Pretest 8 * Post test 8	10	100.0%
Soft CL Daily	Pretest 8 * Post test 8	59	100.0%
Soft CL Biweekly	Pretest 8 * Post test 8	7	100.0%
Soft CL Monthly	Pretest 8 * Post test 8	56	100.0%

Pretest 8 * Post test 8 Crosstabulation

Schedule				Post test 8	Total
RGP permanent	Pretest 8		Count	1	1
			% of Total	20.0%	20.0%
			Count	4	4
			% of Total	80.0%	80.0%
	Total		Count	5	5
			% of Total	100.0%	100.0%
Soft CL Daily	Pretest 8		Count	6	6
			% of Total	12.8%	12.8%
			Count	41	41
			% of Total	87.2%	87.2%
	Total		Count	47	47
			% of Total	100.0%	100.0%
Soft CL Biweekly	Pretest 8		Count	5	5
			% of Total	100.0%	100.0%
	Total		Count	5	5
			% of Total	100.0%	100.0%
Soft CL Monthly	Pretest 8		Count	25	25
			% of Total	51.0%	51.0%
			Count	24	24
			% of Total	49.0%	49.0%
	Total		Count	49	49
			% of Total	100.0%	100.0%

Chi-Square Tests

Schedule		Value	df	Asymptotic Significance (2-sided)
RGP permanent	McNemar-Bowker Test	.	.	.[a]
	N of Valid Cases	5		
Soft CL Daily	McNemar-Bowker Test	.	.	.[a]
	N of Valid Cases	47		
Soft CL Biweekly	McNemar-Bowker Test	.	.	.[a]
	N of Valid Cases	5		
Soft CL Monthly	McNemar-Bowker Test	.	.	.[a]
	N of Valid Cases	49		

a. Computed only for a PxP table, where P must be greater than 1.

Crosstabs

Notes

Output Created		09-SEP-2022 16:26:56
Comments		
Input	Data	/Users/Jacky/Desktop/SPSS CL modality/PrePostTest_Behavior.sav
	Active Dataset	DataSet1
	Filter	<none>
	Weight	<none>
	Split File	Schedule
	N of Rows in Working Data File	132
Missing Value Handling	Definition of Missing	User-defined missing values are treated as missing.
	Cases Used	Statistics for each table are based on all the cases with valid data in the specified range(s) for all variables in each table.
Syntax		CROSSTABS /TABLES=Pretest10 BY Posttest10 /FORMAT=AVALUE TABLES /STATISTICS=MCNEMAR /CELLS=COUNT TOTAL /COUNT ROUND CELL.
Resources	Processor Time	00:00:00.02
	Elapsed Time	00:00:00.00
	Dimensions Requested	2
	Cells Available	524245

Warnings

No measures of association are computed for the crosstabulation of Pretest 10 * Post test 10 for split file Schedule=RGP permanent. At least one variable in each 2-way table upon which measures of association are computed is a constant.
No measures of association are computed for the crosstabulation of Pretest 10 * Post test 10 for split file Schedule=Soft CL Daily. At least one variable in each 2-way table upon which measures of association are computed is a constant.
No measures of association are computed for the crosstabulation of Pretest 10 * Post test 10 for split file Schedule=Soft CL Biweekly. At least one variable in each 2-way table upon which measures of association are computed is a constant.
No measures of association are computed for the crosstabulation of Pretest 10 * Post test 10 for split file Schedule=Soft CL Monthly. At least one variable in each 2-way table upon which measures of association are computed is a constant.

Case Processing Summary

Schedule		Cases			
		Valid		Missing	
		N	Percent	N	Percent
RGP permanent	Pretest 10 * Post test 10	5	50.0%	5	50.0%
Soft CL Daily	Pretest 10 * Post test 10	47	79.7%	12	20.3%
Soft CL Biweekly	Pretest 10 * Post test 10	5	71.4%	2	28.6%
Soft CL Monthly	Pretest 10 * Post test 10	49	87.5%	7	12.5%

Case Processing Summary

Schedule		Cases	
		Total	
		N	Percent
RGP permanent	Pretest 10 * Post test 10	10	100.0%
Soft CL Daily	Pretest 10 * Post test 10	59	100.0%
Soft CL Biweekly	Pretest 10 * Post test 10	7	100.0%
Soft CL Monthly	Pretest 10 * Post test 10	56	100.0%

Pretest 10 * Post test 10 Crosstabulation

Schedule			Post test 10	Total
RGP permanent	Pretest 10	Count	1	1
		% of Total	20.0%	20.0%
		Count	4	4
		% of Total	80.0%	80.0%
	Total	Count	5	5
		% of Total	100.0%	100.0%
Soft CL Daily	Pretest 10	Count	3	3
		% of Total	6.4%	6.4%
		Count	44	44
		% of Total	93.6%	93.6%
	Total	Count	47	47
		% of Total	100.0%	100.0%
Soft CL Biweekly	Pretest 10	Count	1	1
		% of Total	20.0%	20.0%
		Count	4	4
		% of Total	80.0%	80.0%
	Total	Count	5	5
		% of Total	100.0%	100.0%
Soft CL Monthly	Pretest 10	Count	4	4
		% of Total	8.2%	8.2%
		Count	45	45
		% of Total	91.8%	91.8%
	Total	Count	49	49
		% of Total	100.0%	100.0%

Chi-Square Tests

Schedule		Value	df	Asymptotic Significance (2-sided)
RGP permanent	McNemar-Bowker Test	.	.	.[a]
	N of Valid Cases	5		
Soft CL Daily	McNemar-Bowker Test	.	.	.[a]
	N of Valid Cases	47		
Soft CL Biweekly	McNemar-Bowker Test	.	.	.[a]
	N of Valid Cases	5		
Soft CL Monthly	McNemar-Bowker Test	.	.	.[a]
	N of Valid Cases	49		

a. Computed only for a PxP table, where P must be greater than 1.

Crosstabs

Notes

Output Created		09-SEP-2022 16:27:54
Comments		
Input	Data	/Users/Jacky/Desktop/SPSS CL modality/PrePostTest_Behavior.sav
	Active Dataset	DataSet1
	Filter	<none>
	Weight	<none>
	Split File	Schedule
	N of Rows in Working Data File	132
Missing Value Handling	Definition of Missing	User-defined missing values are treated as missing.
	Cases Used	Statistics for each table are based on all the cases with valid data in the specified range(s) for all variables in each table.
Syntax		CROSSTABS /TABLES=Pretest11 BY Posttest11 /FORMAT=AVALUE TABLES /STATISTICS=MCNEMAR /CELLS=COUNT TOTAL /COUNT ROUND CELL.
Resources	Processor Time	00:00:00.02
	Elapsed Time	00:00:00.00
	Dimensions Requested	2
	Cells Available	524245

Warnings

No measures of association are computed for the crosstabulation of Pretest 11 * Posttest 11 for split file Schedule=RGP permanent. At least one variable in each 2-way table upon which measures of association are computed is a constant.
No measures of association are computed for the crosstabulation of Pretest 11 * Posttest 11 for split file Schedule=Soft CL Daily. At least one variable in each 2-way table upon which measures of association are computed is a constant.
No measures of association are computed for the crosstabulation of Pretest 11 * Posttest 11 for split file Schedule=Soft CL Biweekly. At least one variable in each 2-way table upon which measures of association are computed is a constant.
No measures of association are computed for the crosstabulation of Pretest 11 * Posttest 11 for split file Schedule=Soft CL Monthly. At least one variable in each 2-way table upon which measures of association are computed is a constant.

Case Processing Summary

		Cases			
		Valid		Missing	
Schedule		N	Percent	N	Percent
RGP permanent	Pretest 11 * Posttest 11	5	50.0%	5	50.0%
Soft CL Daily	Pretest 11 * Posttest 11	47	79.7%	12	20.3%
Soft CL Biweekly	Pretest 11 * Posttest 11	5	71.4%	2	28.6%
Soft CL Monthly	Pretest 11 * Posttest 11	49	87.5%	7	12.5%

Case Processing Summary

		Cases	
		Total	
Schedule		N	Percent
RGP permanent	Pretest 11 * Posttest 11	10	100.0%
Soft CL Daily	Pretest 11 * Posttest 11	59	100.0%
Soft CL Biweekly	Pretest 11 * Posttest 11	7	100.0%
Soft CL Monthly	Pretest 11 * Posttest 11	56	100.0%

Pretest 11 * Posttest 11 Crosstabulation

Schedule			Posttest 11	Total
RGP permanent	Pretest 11	Count	1	1
		% of Total	20.0%	20.0%
		Count	4	4
		% of Total	80.0%	80.0%
	Total	Count	5	5
		% of Total	100.0%	100.0%
Soft CL Daily	Pretest 11	Count	3	3
		% of Total	6.4%	6.4%
		Count	44	44
		% of Total	93.6%	93.6%
	Total	Count	47	47
		% of Total	100.0%	100.0%
Soft CL Biweekly	Pretest 11	Count	1	1
		% of Total	20.0%	20.0%
		Count	4	4
		% of Total	80.0%	80.0%
	Total	Count	5	5
		% of Total	100.0%	100.0%
Soft CL Monthly	Pretest 11	Count	1	1
		% of Total	2.0%	2.0%
		Count	48	48
		% of Total	98.0%	98.0%
	Total	Count	49	49
		% of Total	100.0%	100.0%

Chi-Square Tests

Schedule		Value	df	Asymptotic Significance (2-sided)
RGP permanent	McNemar-Bowker Test	.	.	.[a]
	N of Valid Cases	5		
Soft CL Daily	McNemar-Bowker Test	.	.	.[a]
	N of Valid Cases	47		
Soft CL Biweekly	McNemar-Bowker Test	.	.	.[a]
	N of Valid Cases	5		
Soft CL Monthly	McNemar-Bowker Test	.	.	.[a]
	N of Valid Cases	49		

a. Computed only for a PxP table, where P must be greater than 1.

Crosstabs

Notes

Output Created		09-SEP-2022 16:28:13
Comments		
Input	Data	/Users/Jacky/Desktop/SPSS CL modality/PrePostTest_Behavior.sav
	Active Dataset	DataSet1
	Filter	<none>
	Weight	<none>
	Split File	Schedule
	N of Rows in Working Data File	132
Missing Value Handling	Definition of Missing	User-defined missing values are treated as missing.
	Cases Used	Statistics for each table are based on all the cases with valid data in the specified range(s) for all variables in each table.
Syntax		CROSSTABS /TABLES=Pretest13 BY Posttest13 /FORMAT=AVALUE TABLES /STATISTICS=MCNEMAR /CELLS=COUNT TOTAL /COUNT ROUND CELL.
Resources	Processor Time	00:00:00.02
	Elapsed Time	00:00:00.00
	Dimensions Requested	2
	Cells Available	524245

Warnings

No measures of association are computed for the crosstabulation of Pretest 13 * Posttest 13 for split file Schedule=RGP permanent. At least one variable in each 2-way table upon which measures of association are computed is a constant.
No measures of association are computed for the crosstabulation of Pretest 13 * Posttest 13 for split file Schedule=Soft CL Biweekly. At least one variable in each 2-way table upon which measures of association are computed is a constant.

Case Processing Summary

Schedule		Cases			
		Valid		Missing	
		N	Percent	N	Percent
RGP permanent	Pretest 13 * Posttest 13	5	50.0%	5	50.0%
Soft CL Daily	Pretest 13 * Posttest 13	47	79.7%	12	20.3%
Soft CL Biweekly	Pretest 13 * Posttest 13	5	71.4%	2	28.6%
Soft CL Monthly	Pretest 13 * Posttest 13	49	87.5%	7	12.5%

Case Processing Summary

Schedule		Cases	
		Total	
		N	Percent
RGP permanent	Pretest 13 * Posttest 13	10	100.0%
Soft CL Daily	Pretest 13 * Posttest 13	59	100.0%
Soft CL Biweekly	Pretest 13 * Posttest 13	7	100.0%
Soft CL Monthly	Pretest 13 * Posttest 13	56	100.0%

Pretest 13 * Posttest 13 Crosstabulation

Schedule			Posttest 13		Total
RGP permanent	Pretest 13	Count		1	1
		% of Total		20.0%	20.0%
		Count		4	4
		% of Total		80.0%	80.0%
	Total	Count		5	5
		% of Total		100.0%	100.0%
Soft CL Daily	Pretest 13	Count	0	6	6
		% of Total	0.0%	12.8%	12.8%
		Count	1	40	41
		% of Total	2.1%	85.1%	87.2%
	Total	Count	1	46	47
		% of Total	2.1%	97.9%	100.0%
Soft CL Biweekly	Pretest 13	Count		3	3
		% of Total		60.0%	60.0%
		Count		2	2
		% of Total		40.0%	40.0%
	Total	Count		5	5
		% of Total		100.0%	100.0%
Soft CL Monthly	Pretest 13	Count	1	17	18
		% of Total	2.0%	34.7%	36.7%
		Count	2	29	31
		% of Total	4.1%	59.2%	63.3%
	Total	Count	3	46	49
		% of Total	6.1%	93.9%	100.0%

Chi-Square Tests

Schedule		Value	df	Asymptotic Significance (2-sided)	Exact Sig. (2-sided)
RGP permanent	McNemar-Bowker Test	.	.	.[a]	
	N of Valid Cases	5			
Soft CL Daily	N of Valid Cases	47			
	McNemar Test				.125[b]
Soft CL Biweekly	McNemar-Bowker Test	.	.	.[a]	
	N of Valid Cases	5			
Soft CL Monthly	N of Valid Cases	49			
	McNemar Test				<.001[b]

a. Computed only for a PxP table, where P must be greater than 1.

b. Binomial distribution used.

Crosstabs

Notes

Output Created		09-SEP-2022 16:28:26
Comments		
Input	Data	/Users/Jacky/Desktop/SPSS CL modality/PrePostTest_Behavior.sav
	Active Dataset	DataSet1
	Filter	<none>
	Weight	<none>
	Split File	Schedule
	N of Rows in Working Data File	132
Missing Value Handling	Definition of Missing	User-defined missing values are treated as missing.
	Cases Used	Statistics for each table are based on all the cases with valid data in the specified range(s) for all variables in each table.
Syntax		CROSSTABS /TABLES=Pretest14 BY Posttest14 /FORMAT=AVALUE TABLES /STATISTICS=MCNEMAR /CELLS=COUNT TOTAL /COUNT ROUND CELL.
Resources	Processor Time	00:00:00.02
	Elapsed Time	00:00:00.00
	Dimensions Requested	2
	Cells Available	524245

Warnings

No measures of association are computed for the crosstabulation of Pretest 14 * Posttest 14 for split file Schedule=RGP permanent. At least one variable in each 2-way table upon which measures of association are computed is a constant.
No measures of association are computed for the crosstabulation of Pretest 14 * Posttest 14 for split file Schedule=Soft CL Biweekly. At least one variable in each 2-way table upon which measures of association are computed is a constant.
No measures of association are computed for the crosstabulation of Pretest 14 * Posttest 14 for split file Schedule=Soft CL Monthly. At least one variable in each 2-way table upon which measures of association are computed is a constant.

Case Processing Summary

		Cases			
		Valid		Missing	
Schedule		N	Percent	N	Percent
RGP permanent	Pretest 14 * Posttest 14	5	50.0%	5	50.0%
Soft CL Daily	Pretest 14 * Posttest 14	47	79.7%	12	20.3%
Soft CL Biweekly	Pretest 14 * Posttest 14	5	71.4%	2	28.6%
Soft CL Monthly	Pretest 14 * Posttest 14	49	87.5%	7	12.5%

Case Processing Summary

		Cases	
		Total	
Schedule		N	Percent
RGP permanent	Pretest 14 * Posttest 14	10	100.0%
Soft CL Daily	Pretest 14 * Posttest 14	59	100.0%
Soft CL Biweekly	Pretest 14 * Posttest 14	7	100.0%
Soft CL Monthly	Pretest 14 * Posttest 14	56	100.0%

Pretest 14 * Posttest 14 Crosstabulation

Schedule			Posttest 14		Total
RGP permanent	Pretest 14	Count		1	1
		% of Total		20.0%	20.0%
		Count		4	4
		% of Total		80.0%	80.0%
	Total	Count		5	5
		% of Total		100.0%	100.0%
Soft CL Daily	Pretest 14	Count	0	14	14
		% of Total	0.0%	29.8%	29.8%
		Count	1	32	33
		% of Total	2.1%	68.1%	70.2%
	Total	Count	1	46	47
		% of Total	2.1%	97.9%	100.0%
Soft CL Biweekly	Pretest 14	Count		2	2
		% of Total		40.0%	40.0%
		Count		3	3
		% of Total		60.0%	60.0%
	Total	Count		5	5
		% of Total		100.0%	100.0%
Soft CL Monthly	Pretest 14	Count		20	20
		% of Total		40.8%	40.8%
		Count		29	29
		% of Total		59.2%	59.2%
	Total	Count		49	49
		% of Total		100.0%	100.0%

Chi-Square Tests

Schedule		Value	df	Asymptotic Significance (2-sided)	Exact Sig. (2-sided)
RGP permanent	McNemar-Bowker Test	.	.	.[a]	
	N of Valid Cases	5			
Soft CL Daily	N of Valid Cases	47			
	McNemar Test				<.001[b]
Soft CL Biweekly	McNemar-Bowker Test	.	.	.[a]	
	N of Valid Cases	5			
Soft CL Monthly	McNemar-Bowker Test	.	.	.[a]	
	N of Valid Cases	49			

a. Computed only for a PxP table, where P must be greater than 1.

b. Binomial distribution used.

Crosstabs

Notes

Output Created		09-SEP-2022 16:28:42
Comments		
Input	Data	/Users/Jacky/Desktop/SPSS CL modality/PrePostTest_Behavior.sav
	Active Dataset	DataSet1
	Filter	<none>
	Weight	<none>
	Split File	Schedule
	N of Rows in Working Data File	132
Missing Value Handling	Definition of Missing	User-defined missing values are treated as missing.
	Cases Used	Statistics for each table are based on all the cases with valid data in the specified range(s) for all variables in each table.
Syntax		CROSSTABS /TABLES=Pretest15 BY Posttest15 /FORMAT=AVALUE TABLES /STATISTICS=MCNEMAR /CELLS=COUNT TOTAL /COUNT ROUND CELL.
Resources	Processor Time	00:00:00.02
	Elapsed Time	00:00:00.00
	Dimensions Requested	2
	Cells Available	524245

Warnings

No measures of association are computed for the crosstabulation of Pretest 15 * Posttest 15 for split file Schedule=RGP permanent. At least one variable in each 2-way table upon which measures of association are computed is a constant.
No measures of association are computed for the crosstabulation of Pretest 15 * Posttest 15 for split file Schedule=Soft CL Daily. At least one variable in each 2-way table upon which measures of association are computed is a constant.
No measures of association are computed for the crosstabulation of Pretest 15 * Posttest 15 for split file Schedule=Soft CL Biweekly. At least one variable in each 2-way table upon which measures of association are computed is a constant.
No measures of association are computed for the crosstabulation of Pretest 15 * Posttest 15 for split file Schedule=Soft CL Monthly. At least one variable in each 2-way table upon which measures of association are computed is a constant.

Case Processing Summary

		Cases			
		Valid		Missing	
Schedule		N	Percent	N	Percent
RGP permanent	Pretest 15 * Posttest 15	5	50.0%	5	50.0%
Soft CL Daily	Pretest 15 * Posttest 15	47	79.7%	12	20.3%
Soft CL Biweekly	Pretest 15 * Posttest 15	5	71.4%	2	28.6%
Soft CL Monthly	Pretest 15 * Posttest 15	49	87.5%	7	12.5%

Case Processing Summary

		Cases	
		Total	
Schedule		N	Percent
RGP permanent	Pretest 15 * Posttest 15	10	100.0%
Soft CL Daily	Pretest 15 * Posttest 15	59	100.0%
Soft CL Biweekly	Pretest 15 * Posttest 15	7	100.0%
Soft CL Monthly	Pretest 15 * Posttest 15	56	100.0%

Pretest 15 * Posttest 15 Crosstabulation

Schedule			Posttest 15	Total
RGP permanent	Pretest 15	Count	3	3
		% of Total	60.0%	60.0%
		Count	2	2
		% of Total	40.0%	40.0%
	Total	Count	5	5
		% of Total	100.0%	100.0%
Soft CL Daily	Pretest 15	Count	28	28
		% of Total	59.6%	59.6%
		Count	19	19
		% of Total	40.4%	40.4%
	Total	Count	47	47
		% of Total	100.0%	100.0%
Soft CL Biweekly	Pretest 15	Count	4	4
		% of Total	80.0%	80.0%
		Count	1	1
		% of Total	20.0%	20.0%
	Total	Count	5	5
		% of Total	100.0%	100.0%
Soft CL Monthly	Pretest 15	Count	23	23
		% of Total	46.9%	46.9%
		Count	26	26
		% of Total	53.1%	53.1%
	Total	Count	49	49
		% of Total	100.0%	100.0%

Chi-Square Tests

Schedule		Value	df	Asymptotic Significance (2-sided)
RGP permanent	McNemar-Bowker Test	.	.	.[a]
	N of Valid Cases	5		
Soft CL Daily	McNemar-Bowker Test	.	.	.[a]
	N of Valid Cases	47		
Soft CL Biweekly	McNemar-Bowker Test	.	.	.[a]
	N of Valid Cases	5		
Soft CL Monthly	McNemar-Bowker Test	.	.	.[a]
	N of Valid Cases	49		

a. Computed only for a PxP table, where P must be greater than 1.

Crosstabs

Notes

Output Created		09-SEP-2022 16:29:01
Comments		
Input	Data	/Users/Jacky/Desktop/SPSS CL modality/PrePostTest_Behavior.sav
	Active Dataset	DataSet1
	Filter	<none>
	Weight	<none>
	Split File	Schedule
	N of Rows in Working Data File	132
Missing Value Handling	Definition of Missing	User-defined missing values are treated as missing.
	Cases Used	Statistics for each table are based on all the cases with valid data in the specified range(s) for all variables in each table.
Syntax		CROSSTABS /TABLES=Pretest17 BY Posttest17 /FORMAT=AVALUE TABLES /STATISTICS=MCNEMAR /CELLS=COUNT TOTAL /COUNT ROUND CELL.
Resources	Processor Time	00:00:00.02
	Elapsed Time	00:00:00.00
	Dimensions Requested	2
	Cells Available	524245

Warnings

No measures of association are computed for the crosstabulation of Pretest 17 * Posttest 17 for split file Schedule=RGP permanent. At least one variable in each 2-way table upon which measures of association are computed is a constant.
No measures of association are computed for the crosstabulation of Pretest 17 * Posttest 17 for split file Schedule=Soft CL Daily. At least one variable in each 2-way table upon which measures of association are computed is a constant.
No measures of association are computed for the crosstabulation of Pretest 17 * Posttest 17 for split file Schedule=Soft CL Biweekly. At least one variable in each 2-way table upon which measures of association are computed is a constant.
No measures of association are computed for the crosstabulation of Pretest 17 * Posttest 17 for split file Schedule=Soft CL Monthly. At least one variable in each 2-way table upon which measures of association are computed is a constant.

Case Processing Summary

		Cases			
		Valid		Missing	
Schedule		N	Percent	N	Percent
RGP permanent	Pretest 17 * Posttest 17	5	50.0%	5	50.0%
Soft CL Daily	Pretest 17 * Posttest 17	47	79.7%	12	20.3%
Soft CL Biweekly	Pretest 17 * Posttest 17	5	71.4%	2	28.6%
Soft CL Monthly	Pretest 17 * Posttest 17	49	87.5%	7	12.5%

Case Processing Summary

		Cases	
		Total	
Schedule		N	Percent
RGP permanent	Pretest 17 * Posttest 17	10	100.0%
Soft CL Daily	Pretest 17 * Posttest 17	59	100.0%
Soft CL Biweekly	Pretest 17 * Posttest 17	7	100.0%
Soft CL Monthly	Pretest 17 * Posttest 17	56	100.0%

Pretest 17 * Posttest 17 Crosstabulation

Schedule			Posttest 17	Total
RGP permanent	Pretest 17	Count	2	2
		% of Total	40.0%	40.0%
		Count	3	3
		% of Total	60.0%	60.0%
	Total	Count	5	5
		% of Total	100.0%	100.0%
Soft CL Daily	Pretest 17	Count	6	6
		% of Total	12.8%	12.8%
		Count	41	41
		% of Total	87.2%	87.2%
	Total	Count	47	47
		% of Total	100.0%	100.0%
Soft CL Biweekly	Pretest 17	Count	2	2
		% of Total	40.0%	40.0%
		Count	3	3
		% of Total	60.0%	60.0%
	Total	Count	5	5
		% of Total	100.0%	100.0%
Soft CL Monthly	Pretest 17	Count	19	19
		% of Total	38.8%	38.8%
		Count	30	30
		% of Total	61.2%	61.2%
	Total	Count	49	49
		% of Total	100.0%	100.0%

Chi-Square Tests

Schedule		Value	df	Asymptotic Significance (2-sided)
RGP permanent	McNemar-Bowker Test	.	.	.[a]
	N of Valid Cases	5		
Soft CL Daily	McNemar-Bowker Test	.	.	.[a]
	N of Valid Cases	47		
Soft CL Biweekly	McNemar-Bowker Test	.	.	.[a]
	N of Valid Cases	5		
Soft CL Monthly	McNemar-Bowker Test	.	.	.[a]
	N of Valid Cases	49		

a. Computed only for a PxP table, where P must be greater than 1.

Crosstabs

Notes

Output Created		09-SEP-2022 16:29:12
Comments		
Input	Data	/Users/Jacky/Desktop/SPSS CL modality/PrePostTest_Behavior.sav
	Active Dataset	DataSet1
	Filter	<none>
	Weight	<none>
	Split File	Schedule
	N of Rows in Working Data File	132
Missing Value Handling	Definition of Missing	User-defined missing values are treated as missing.
	Cases Used	Statistics for each table are based on all the cases with valid data in the specified range(s) for all variables in each table.
Syntax		CROSSTABS /TABLES=Pretest18 BY Posttest18 /FORMAT=AVALUE TABLES /STATISTICS=MCNEMAR /CELLS=COUNT TOTAL /COUNT ROUND CELL.
Resources	Processor Time	00:00:00.02
	Elapsed Time	00:00:00.00
	Dimensions Requested	2
	Cells Available	524245

Warnings

No measures of association are computed for the crosstabulation of Pretest 18 * Posttest 18 for split file Schedule=RGP permanent. At least one variable in each 2-way table upon which measures of association are computed is a constant.
No measures of association are computed for the crosstabulation of Pretest 18 * Posttest 18 for split file Schedule=Soft CL Daily. At least one variable in each 2-way table upon which measures of association are computed is a constant.
No measures of association are computed for the crosstabulation of Pretest 18 * Posttest 18 for split file Schedule=Soft CL Biweekly. At least one variable in each 2-way table upon which measures of association are computed is a constant.
No measures of association are computed for the crosstabulation of Pretest 18 * Posttest 18 for split file Schedule=Soft CL Monthly. At least one variable in each 2-way table upon which measures of association are computed is a constant.

Case Processing Summary

		Cases			
		Valid		Missing	
Schedule		N	Percent	N	Percent
RGP permanent	Pretest 18 * Posttest 18	5	50.0%	5	50.0%
Soft CL Daily	Pretest 18 * Posttest 18	47	79.7%	12	20.3%
Soft CL Biweekly	Pretest 18 * Posttest 18	5	71.4%	2	28.6%
Soft CL Monthly	Pretest 18 * Posttest 18	49	87.5%	7	12.5%

Case Processing Summary

		Cases	
		Total	
Schedule		N	Percent
RGP permanent	Pretest 18 * Posttest 18	10	100.0%
Soft CL Daily	Pretest 18 * Posttest 18	59	100.0%
Soft CL Biweekly	Pretest 18 * Posttest 18	7	100.0%
Soft CL Monthly	Pretest 18 * Posttest 18	56	100.0%

Pretest 18 * Posttest 18 Crosstabulation

Schedule			Posttest 18	Total
RGP permanent	Pretest 18	Count	2	2
		% of Total	40.0%	40.0%
		Count	3	3
		% of Total	60.0%	60.0%
	Total	Count	5	5
		% of Total	100.0%	100.0%
Soft CL Daily	Pretest 18	Count	7	7
		% of Total	14.9%	14.9%
		Count	40	40
		% of Total	85.1%	85.1%
	Total	Count	47	47
		% of Total	100.0%	100.0%
Soft CL Biweekly	Pretest 18	Count	1	1
		% of Total	20.0%	20.0%
		Count	4	4
		% of Total	80.0%	80.0%
	Total	Count	5	5
		% of Total	100.0%	100.0%
Soft CL Monthly	Pretest 18	Count	20	20
		% of Total	40.8%	40.8%
		Count	29	29
		% of Total	59.2%	59.2%
	Total	Count	49	49
		% of Total	100.0%	100.0%

Chi-Square Tests

Schedule		Value	df	Asymptotic Significance (2-sided)
RGP permanent	McNemar-Bowker Test	.	.	.[a]
	N of Valid Cases	5		
Soft CL Daily	McNemar-Bowker Test	.	.	.[a]
	N of Valid Cases	47		
Soft CL Biweekly	McNemar-Bowker Test	.	.	.[a]
	N of Valid Cases	5		
Soft CL Monthly	McNemar-Bowker Test	.	.	.[a]
	N of Valid Cases	49		

a. Computed only for a PxP table, where P must be greater than 1.

Crosstabs

Notes

Output Created		09-SEP-2022 16:29:26
Comments		
Input	Data	/Users/Jacky/Desktop/SPSS CL modality/PrePostTest_Behavior.sav
	Active Dataset	DataSet1
	Filter	<none>
	Weight	<none>
	Split File	Schedule
	N of Rows in Working Data File	132
Missing Value Handling	Definition of Missing	User-defined missing values are treated as missing.
	Cases Used	Statistics for each table are based on all the cases with valid data in the specified range(s) for all variables in each table.
Syntax		CROSSTABS /TABLES=Pretest19 BY Posttest19 /FORMAT=AVALUE TABLES /STATISTICS=MCNEMAR /CELLS=COUNT TOTAL /COUNT ROUND CELL.
Resources	Processor Time	00:00:00.02
	Elapsed Time	00:00:00.00
	Dimensions Requested	2
	Cells Available	524245

Warnings

No measures of association are computed for the crosstabulation of Pretest 19 * Posttest 19 for split file Schedule=RGP permanent. At least one variable in each 2-way table upon which measures of association are computed is a constant.
No measures of association are computed for the crosstabulation of Pretest 19 * Posttest 19 for split file Schedule=Soft CL Daily. At least one variable in each 2-way table upon which measures of association are computed is a constant.
No measures of association are computed for the crosstabulation of Pretest 19 * Posttest 19 for split file Schedule=Soft CL Biweekly. At least one variable in each 2-way table upon which measures of association are computed is a constant.
No measures of association are computed for the crosstabulation of Pretest 19 * Posttest 19 for split file Schedule=Soft CL Monthly. At least one variable in each 2-way table upon which measures of association are computed is a constant.

Case Processing Summary

		Cases			
		Valid		Missing	
Schedule		N	Percent	N	Percent
RGP permanent	Pretest 19 * Posttest 19	6	60.0%	4	40.0%
Soft CL Daily	Pretest 19 * Posttest 19	44	74.6%	15	25.4%
Soft CL Biweekly	Pretest 19 * Posttest 19	6	85.7%	1	14.3%
Soft CL Monthly	Pretest 19 * Posttest 19	50	89.3%	6	10.7%

Case Processing Summary

		Cases	
		Total	
Schedule		N	Percent
RGP permanent	Pretest 19 * Posttest 19	10	100.0%
Soft CL Daily	Pretest 19 * Posttest 19	59	100.0%
Soft CL Biweekly	Pretest 19 * Posttest 19	7	100.0%
Soft CL Monthly	Pretest 19 * Posttest 19	56	100.0%

Pretest 19 * Posttest 19 Crosstabulation

Schedule			Posttest 19	Total
RGP permanent	Pretest 19	Count	1	1
		% of Total	16.7%	16.7%
		Count	5	5
		% of Total	83.3%	83.3%
	Total	Count	6	6
		% of Total	100.0%	100.0%
Soft CL Daily	Pretest 19	Count	10	10
		% of Total	22.7%	22.7%
		Count	34	34
		% of Total	77.3%	77.3%
	Total	Count	44	44
		% of Total	100.0%	100.0%
Soft CL Biweekly	Pretest 19	Count	4	4
		% of Total	66.7%	66.7%
		Count	2	2
		% of Total	33.3%	33.3%
	Total	Count	6	6
		% of Total	100.0%	100.0%
Soft CL Monthly	Pretest 19	Count	26	26
		% of Total	52.0%	52.0%
		Count	24	24
		% of Total	48.0%	48.0%
	Total	Count	50	50
		% of Total	100.0%	100.0%

Chi-Square Tests

Schedule		Value	df	Asymptotic Significance (2-sided)
RGP permanent	McNemar-Bowker Test	.	.	.[a]
	N of Valid Cases	6		
Soft CL Daily	McNemar-Bowker Test	.	.	.[a]
	N of Valid Cases	44		
Soft CL Biweekly	McNemar-Bowker Test	.	.	.[a]
	N of Valid Cases	6		
Soft CL Monthly	McNemar-Bowker Test	.	.	.[a]
	N of Valid Cases	50		

a. Computed only for a PxP table, where P must be greater than 1.
