# Supplementary material for: Effectiveness of an online educational video intervention to improve the knowledge and behavior of contact lens care during the COVID-19 pandemic: A pre-test/post-test design
Source: Heliyon. 2022 Oct 11;8(10):e11009. doi: 10.1016/j.heliyon.2022.e11009 (PMC9551115; doi:10.1016/j.heliyon.2022.e11009)
Supplement: Knowledge Subgroup Analysis [file mmc2.pdf]

Crosstabs

Notes

Output Created		09-SEP-2022 16:33:05
Comments		
Input	Data	/Users/Jacky/Desktop/SPSS CL modality/PrePostTest_Knowledge1.sav
	Active Dataset	DataSet2
	Filter	<none>
	Weight	<none>
	Split File	Schedule
	N of Rows in Working Data File	132
Missing Value Handling	Definition of Missing	User-defined missing values are treated as missing.
	Cases Used	Statistics for each table are based on all the cases with valid data in the specified range(s) for all variables in each table.
Syntax		CROSSTABS<br>/TABLES=Pretest1 BY Posttest1<br>/FORMAT=AVALUE TABLES<br><br>/STATISTICS=MCNEMAR<br>/CELLS=COUNT<br>/COUNT ROUND CELL.
Resources	Processor Time	00:00:00.02
	Elapsed Time	00:00:00.00
	Dimensions Requested	2
	Cells Available	524245

Warnings

No measures of association are computed for the crosstabulation of Pretest 1 * Post test 1 for split file Schedule=RGP permanent. At least one variable in each 2-way table upon which measures of association are computed is a constant.
No measures of association are computed for the crosstabulation of Pretest 1 * Post test 1 for split file Schedule=Soft CL Biweekly. At least one variable in each 2-way table upon which measures of association are computed is a constant.

Case Processing Summary

Schedule		Cases Valid N	Valid Percent	Missing N	Missing Percent
RGP permanent	Pretest 1 * Post test 1	10	100.0%	0	0.0%
Soft CL Daily	Pretest 1 * Post test 1	59	100.0%	0	0.0%
Soft CL Biweekly	Pretest 1 * Post test 1	7	100.0%	0	0.0%
Soft CL Monthly	Pretest 1 * Post test 1	56	100.0%	0	0.0%

Case Processing Summary

Schedule		Cases Total N	Total Percent
RGP permanent	Pretest 1 * Post test 1	10	100.0%
Soft CL Daily	Pretest 1 * Post test 1	59	100.0%
Soft CL Biweekly	Pretest 1 * Post test 1	7	100.0%
Soft CL Monthly	Pretest 1 * Post test 1	56	100.0%

Pretest 1 * Post test 1 Crosstabulation

Count

Schedule			Post test 1		Total
RGP permanent	Pretest 1			2	2
				8	8
	Total			10	10
Soft CL Daily	Pretest 1		1	21	22
			3	34	37
	Total		4	55	59
Soft CL Biweekly	Pretest 1			7	7
	Total			7	7
Soft CL Monthly	Pretest 1		1	5	6
			3	47	50
	Total		4	52	56

Chi-Square Tests

Schedule		Value	df	Asymptotic Significance (2-sided)	Exact Sig. (2-sided)
RGP permanent	McNemar-Bowker Test	.	.	.[a]	
	N of Valid Cases	10			
Soft CL Daily	N of Valid Cases	59			
	McNemar Test				<.001[b]
Soft CL Biweekly	McNemar-Bowker Test	.	.	.[a]	
	N of Valid Cases	7			
Soft CL Monthly	N of Valid Cases	56			
	McNemar Test				.727[b]

a. Computed only for a PxP table, where P must be greater than 1.

b. Binomial distribution used.

Crosstabs

Notes

Output Created		09-SEP-2022 16:33:39
Comments		
Input	Data	/Users/Jacky/Desktop/SPSS CL modality/PrePostTest_Knowledge1.sav
	Active Dataset	DataSet2
	Filter	<none>
	Weight	<none>
	Split File	Schedule
	N of Rows in Working Data File	132
Missing Value Handling	Definition of Missing	User-defined missing values are treated as missing.
	Cases Used	Statistics for each table are based on all the cases with valid data in the specified range(s) for all variables in each table.
Syntax		CROSSTABS<br>/TABLES=Pretest2 BY Posttest2<br>/FORMAT=AVALUE TABLES<br>/STATISTICS=MCNEMAR<br>/CELLS=COUNT<br>/COUNT ROUND CELL.

Notes

Resources	Processor Time	00:00:00.02
	Elapsed Time	00:00:00.00
	Dimensions Requested	2
	Cells Available	524245

Warnings

No measures of association are computed for the crosstabulation of Pretest 2 * Post test 2 for split file Schedule=RGP permanent. At least one variable in each 2-way table upon which measures of association are computed is a constant.
No measures of association are computed for the crosstabulation of Pretest 2 * Post test 2 for split file Schedule=Soft CL Biweekly. At least one variable in each 2-way table upon which measures of association are computed is a constant.

Case Processing Summary

		Cases			
		Valid		Missing	
Schedule		N	Percent	N	Percent
RGP permanent	Pretest 2 * Post test 2	10	100.0%	0	0.0%
Soft CL Daily	Pretest 2 * Post test 2	59	100.0%	0	0.0%
Soft CL Biweekly	Pretest 2 * Post test 2	7	100.0%	0	0.0%
Soft CL Monthly	Pretest 2 * Post test 2	56	100.0%	0	0.0%

Case Processing Summary

		Cases	
		Total	
Schedule		N	Percent
RGP permanent	Pretest 2 * Post test 2	10	100.0%
Soft CL Daily	Pretest 2 * Post test 2	59	100.0%
Soft CL Biweekly	Pretest 2 * Post test 2	7	100.0%
Soft CL Monthly	Pretest 2 * Post test 2	56	100.0%

Pretest 2 * Post test 2 Crosstabulation

Count

Schedule		Post test 2		Total
RGP permanent	Pretest 2		7	7
			3	3
	Total		10	10
Soft CL Daily	Pretest 2	1	57	58
		0	1	1
	Total	1	58	59
Soft CL Biweekly	Pretest 2		5	5
			2	2
	Total		7	7
Soft CL Monthly	Pretest 2	1	8	9
		5	42	47
	Total	6	50	56

Chi-Square Tests

Schedule		Value	df	Asymptotic Significance (2-sided)	Exact Sig. (2-sided)
RGP permanent	McNemar-Bowker Test	.	.	.[a]	
	N of Valid Cases	10			
Soft CL Daily	N of Valid Cases	59			
	McNemar Test				<.001[b]
Soft CL Biweekly	McNemar-Bowker Test	.	.	.[a]	
	N of Valid Cases	7			
Soft CL Monthly	N of Valid Cases	56			
	McNemar Test				.581[b]

a. Computed only for a PxP table, where P must be greater than 1.

b. Binomial distribution used.

Crosstabs

Notes

Output Created		09-SEP-2022 16:34:00
Comments		
Input	Data	/Users/Jacky/Desktop/SPSS CL modality/PrePostTest_Knowledge1.sav
	Active Dataset	DataSet2
	Filter	<none>
	Weight	<none>
	Split File	Schedule
	N of Rows in Working Data File	132
Missing Value Handling	Definition of Missing	User-defined missing values are treated as missing.
	Cases Used	Statistics for each table are based on all the cases with valid data in the specified range(s) for all variables in each table.
Syntax		CROSSTABS<br>/TABLES=Pretest3 BY Posttest3<br>/FORMAT=AVALUE TABLES<br>/STATISTICS=MCNEMAR<br>/CELLS=COUNT<br>/COUNT ROUND CELL.
Resources	Processor Time	00:00:00.02
	Elapsed Time	00:00:00.00
	Dimensions Requested	2
	Cells Available	524245

Warnings

No measures of association are computed for the crosstabulation of Pretest 3 * Post test 3 for split file Schedule=RGP permanent. At least one variable in each 2-way table upon which measures of association are computed is a constant.
No measures of association are computed for the crosstabulation of Pretest 3 * Post test 3 for split file Schedule=Soft CL Daily. At least one variable in each 2-way table upon which measures of association are computed is a constant.
No measures of association are computed for the crosstabulation of Pretest 3 * Post test 3 for split file Schedule=Soft CL Biweekly. At least one variable in each 2-way table upon which measures of association are computed is a constant.
No measures of association are computed for the crosstabulation of Pretest 3 * Post test 3 for split file Schedule=Soft CL Monthly. At least one variable in each 2-way table upon which measures of association are computed is a constant.

Case Processing Summary

		Cases			
		Valid		Missing	
Schedule		N	Percent	N	Percent
RGP permanent	Pretest 3 * Post test 3	10	100.0%	0	0.0%
Soft CL Daily	Pretest 3 * Post test 3	59	100.0%	0	0.0%
Soft CL Biweekly	Pretest 3 * Post test 3	7	100.0%	0	0.0%
Soft CL Monthly	Pretest 3 * Post test 3	56	100.0%	0	0.0%

Case Processing Summary

		Cases	
		Total	
Schedule		N	Percent
RGP permanent	Pretest 3 * Post test 3	10	100.0%
Soft CL Daily	Pretest 3 * Post test 3	59	100.0%
Soft CL Biweekly	Pretest 3 * Post test 3	7	100.0%
Soft CL Monthly	Pretest 3 * Post test 3	56	100.0%

Pretest 3 * Post test 3 Crosstabulation

Count

Schedule		Post test 3	Total
RGP permanent	Pretest 3	5	5
		5	5
	Total	10	10
Soft CL Daily	Pretest 3	59	59
	Total	59	59
Soft CL Biweekly	Pretest 3	7	7
	Total	7	7
Soft CL Monthly	Pretest 3	26	26
		30	30
	Total	56	56

Chi-Square Tests

Schedule		Value	df	Asymptotic Significance (2-sided)
RGP permanent	McNemar-Bowker Test	.	.	.[a]
	N of Valid Cases	10		
Soft CL Daily	McNemar-Bowker Test	.	.	.[a]
	N of Valid Cases	59		
Soft CL Biweekly	McNemar-Bowker Test	.	.	.[a]
	N of Valid Cases	7		
Soft CL Monthly	McNemar-Bowker Test	.	.	.[a]
	N of Valid Cases	56		

a. Computed only for a PxP table, where P must be greater than 1.

Crosstabs

Notes

Output Created		09-SEP-2022 16:34:15
Comments		
Input	Data	/Users/Jacky/Desktop/SPSS CL modality/PrePostTest_Knowledge1.sav
	Active Dataset	DataSet2
	Filter	<none>
	Weight	<none>
	Split File	Schedule
	N of Rows in Working Data File	132
Missing Value Handling	Definition of Missing	User-defined missing values are treated as missing.
	Cases Used	Statistics for each table are based on all the cases with valid data in the specified range(s) for all variables in each table.
Syntax		CROSSTABS<br>/TABLES=Pretest4 BY Posttest4<br>/FORMAT=AVALUE TABLES<br>/STATISTICS=MCNEMAR<br>/CELLS=COUNT<br>/COUNT ROUND CELL.
Resources	Processor Time	00:00:00.02
	Elapsed Time	00:00:00.00
	Dimensions Requested	2
	Cells Available	524245

Warnings

No measures of association are computed for the crosstabulation of Pretest 4 * Post test 4 for split file Schedule=RGP permanent. At least one variable in each 2-way table upon which measures of association are computed is a constant.
No measures of association are computed for the crosstabulation of Pretest 4 * Post test 4 for split file Schedule=Soft CL Daily. At least one variable in each 2-way table upon which measures of association are computed is a constant.
No measures of association are computed for the crosstabulation of Pretest 4 * Post test 4 for split file Schedule=Soft CL Biweekly. At least one variable in each 2-way table upon which measures of association are computed is a constant.
No measures of association are computed for the crosstabulation of Pretest 4 * Post test 4 for split file Schedule=Soft CL Monthly. At least one variable in each 2-way table upon which measures of association are computed is a constant.

Case Processing Summary

		Cases			
		Valid		Missing	
Schedule		N	Percent	N	Percent
RGP permanent	Pretest 4 * Post test 4	10	100.0%	0	0.0%
Soft CL Daily	Pretest 4 * Post test 4	59	100.0%	0	0.0%
Soft CL Biweekly	Pretest 4 * Post test 4	7	100.0%	0	0.0%
Soft CL Monthly	Pretest 4 * Post test 4	56	100.0%	0	0.0%

Case Processing Summary

		Cases	
		Total	
Schedule		N	Percent
RGP permanent	Pretest 4 * Post test 4	10	100.0%
Soft CL Daily	Pretest 4 * Post test 4	59	100.0%
Soft CL Biweekly	Pretest 4 * Post test 4	7	100.0%
Soft CL Monthly	Pretest 4 * Post test 4	56	100.0%

Pretest 4 * Post test 4 Crosstabulation

Count

Schedule			Post test 4	Total
RGP permanent	Pretest 4		7	7
			3	3
	Total		10	10
Soft CL Daily	Pretest 4		2	2
			57	57
	Total		59	59
Soft CL Biweekly	Pretest 4		2	2
			5	5
	Total		7	7
Soft CL Monthly	Pretest 4		39	39
			17	17
	Total		56	56

Chi-Square Tests

Schedule		Value	df	Asymptotic Significance (2-sided)
RGP permanent	McNemar-Bowker Test	.	.	.[a]
	N of Valid Cases	10		
Soft CL Daily	McNemar-Bowker Test	.	.	.[a]
	N of Valid Cases	59		
Soft CL Biweekly	McNemar-Bowker Test	.	.	.[a]
	N of Valid Cases	7		
Soft CL Monthly	McNemar-Bowker Test	.	.	.[a]
	N of Valid Cases	56		

a. Computed only for a PxP table, where P must be greater than 1.

Crosstabs

Notes

Output Created		09-SEP-2022 16:34:30
Comments		
Input	Data	/Users/Jacky/Desktop/SPSS CL modality/PrePostTest_Knowledge1.sav
	Active Dataset	DataSet2
	Filter	<none>
	Weight	<none>
	Split File	Schedule
	N of Rows in Working Data File	132
Missing Value Handling	Definition of Missing	User-defined missing values are treated as missing.
	Cases Used	Statistics for each table are based on all the cases with valid data in the specified range(s) for all variables in each table.
Syntax		CROSSTABS<br>/TABLES=Pretest5 BY Posttest5<br>/FORMAT=AVALUE TABLES<br>/STATISTICS=MCNEMAR<br>/CELLS=COUNT<br>/COUNT ROUND CELL.
Resources	Processor Time	00:00:00.02
	Elapsed Time	00:00:00.00
	Dimensions Requested	2
	Cells Available	524245

Warnings

No measures of association are computed for the crosstabulation of Pretest 5 * Post test 5 for split file Schedule=RGP permanent. At least one variable in each 2-way table upon which measures of association are computed is a constant.
No measures of association are computed for the crosstabulation of Pretest 5 * Post test 5 for split file Schedule=Soft CL Daily. At least one variable in each 2-way table upon which measures of association are computed is a constant.
No measures of association are computed for the crosstabulation of Pretest 5 * Post test 5 for split file Schedule=Soft CL Biweekly. At least one variable in each 2-way table upon which measures of association are computed is a constant.
No measures of association are computed for the crosstabulation of Pretest 5 * Post test 5 for split file Schedule=Soft CL Monthly. At least one variable in each 2-way table upon which measures of association are computed is a constant.

Case Processing Summary

		Cases			
		Valid		Missing	
Schedule		N	Percent	N	Percent
RGP permanent	Pretest 5 * Post test 5	10	100.0%	0	0.0%
Soft CL Daily	Pretest 5 * Post test 5	59	100.0%	0	0.0%
Soft CL Biweekly	Pretest 5 * Post test 5	7	100.0%	0	0.0%
Soft CL Monthly	Pretest 5 * Post test 5	56	100.0%	0	0.0%

Case Processing Summary

		Cases	
		Total	
Schedule		N	Percent
RGP permanent	Pretest 5 * Post test 5	10	100.0%
Soft CL Daily	Pretest 5 * Post test 5	59	100.0%
Soft CL Biweekly	Pretest 5 * Post test 5	7	100.0%
Soft CL Monthly	Pretest 5 * Post test 5	56	100.0%

Pretest 5 * Post test 5 Crosstabulation

Count

Schedule			Post test 5	Total
RGP permanent	Pretest 5		1	1
			9	9
	Total		10	10
Soft CL Daily	Pretest 5		3	3
			56	56
	Total		59	59
Soft CL Biweekly	Pretest 5		7	7
	Total		7	7
Soft CL Monthly	Pretest 5		27	27
			29	29
	Total		56	56

Chi-Square Tests

Schedule		Value	df	Asymptotic Significance (2-sided)
RGP permanent	McNemar-Bowker Test	.	.	.[a]
	N of Valid Cases	10		
Soft CL Daily	McNemar-Bowker Test	.	.	.[a]
	N of Valid Cases	59		
Soft CL Biweekly	McNemar-Bowker Test	.	.	.[a]
	N of Valid Cases	7		
Soft CL Monthly	McNemar-Bowker Test	.	.	.[a]
	N of Valid Cases	56		

a. Computed only for a PxP table, where P must be greater than 1.

Crosstabs

Notes

Output Created		09-SEP-2022 16:34:41
Comments		
Input	Data	/Users/Jacky/Desktop/SPSS CL modality/PrePostTest_Knowledge1.sav
	Active Dataset	DataSet2
	Filter	<none>
	Weight	<none>
	Split File	Schedule
	N of Rows in Working Data File	132
Missing Value Handling	Definition of Missing	User-defined missing values are treated as missing.
	Cases Used	Statistics for each table are based on all the cases with valid data in the specified range(s) for all variables in each table.
Syntax		CROSSTABS<br>/TABLES=Pretest6 BY Posttest6<br>/FORMAT=AVALUE TABLES<br>/STATISTICS=MCNEMAR<br>/CELLS=COUNT<br>/COUNT ROUND CELL.
Resources	Processor Time	00:00:00.02
	Elapsed Time	00:00:00.00
	Dimensions Requested	2
	Cells Available	524245

Warnings

No measures of association are computed for the crosstabulation of Pretest 6 * Post test 6 for split file Schedule=RGP permanent. At least one variable in each 2-way table upon which measures of association are computed is a constant.
No measures of association are computed for the crosstabulation of Pretest 6 * Post test 6 for split file Schedule=Soft CL Daily. At least one variable in each 2-way table upon which measures of association are computed is a constant.
No measures of association are computed for the crosstabulation of Pretest 6 * Post test 6 for split file Schedule=Soft CL Biweekly. At least one variable in each 2-way table upon which measures of association are computed is a constant.
No measures of association are computed for the crosstabulation of Pretest 6 * Post test 6 for split file Schedule=Soft CL Monthly. At least one variable in each 2-way table upon which measures of association are computed is a constant.

Case Processing Summary

		Cases			
		Valid		Missing	
Schedule		N	Percent	N	Percent
RGP permanent	Pretest 6 * Post test 6	10	100.0%	0	0.0%
Soft CL Daily	Pretest 6 * Post test 6	59	100.0%	0	0.0%
Soft CL Biweekly	Pretest 6 * Post test 6	7	100.0%	0	0.0%
Soft CL Monthly	Pretest 6 * Post test 6	56	100.0%	0	0.0%

Case Processing Summary

		Cases	
		Total	
Schedule		N	Percent
RGP permanent	Pretest 6 * Post test 6	10	100.0%
Soft CL Daily	Pretest 6 * Post test 6	59	100.0%
Soft CL Biweekly	Pretest 6 * Post test 6	7	100.0%
Soft CL Monthly	Pretest 6 * Post test 6	56	100.0%

Pretest 6 * Post test 6 Crosstabulation

Count

Schedule			Post test 6	Total
RGP permanent	Pretest 6		3	3
			7	7
	Total		10	10
Soft CL Daily	Pretest 6		3	3
			56	56
	Total		59	59
Soft CL Biweekly	Pretest 6		1	1
			6	6
	Total		7	7
Soft CL Monthly	Pretest 6		10	10
			46	46
	Total		56	56

Chi-Square Tests

Schedule		Value	df	Asymptotic Significance (2-sided)
RGP permanent	McNemar-Bowker Test	.	.	.[a]
	N of Valid Cases	10		
Soft CL Daily	McNemar-Bowker Test	.	.	.[a]
	N of Valid Cases	59		
Soft CL Biweekly	McNemar-Bowker Test	.	.	.[a]
	N of Valid Cases	7		
Soft CL Monthly	McNemar-Bowker Test	.	.	.[a]
	N of Valid Cases	56		

a. Computed only for a PxP table, where P must be greater than 1.

Crosstabs

Notes

Output Created		09-SEP-2022 16:34:52
Comments		
Input	Data	/Users/Jacky/Desktop/SPSS CL modality/PrePostTest_Knowledge1.sav
	Active Dataset	DataSet2
	Filter	<none>
	Weight	<none>
	Split File	Schedule
	N of Rows in Working Data File	132
Missing Value Handling	Definition of Missing	User-defined missing values are treated as missing.
	Cases Used	Statistics for each table are based on all the cases with valid data in the specified range(s) for all variables in each table.
Syntax		CROSSTABS /TABLES=Pretest7 BY Posttest7 /FORMAT=AVALUE TABLES /STATISTICS=MCNEMAR /CELLS=COUNT /COUNT ROUND CELL.
Resources	Processor Time	00:00:00.02
	Elapsed Time	00:00:00.00
	Dimensions Requested	2
	Cells Available	524245

Warnings

No measures of association are computed for the crosstabulation of Pretest 7 * Post test 7 for split file Schedule=RGP permanent. At least one variable in each 2-way table upon which measures of association are computed is a constant.
No measures of association are computed for the crosstabulation of Pretest 7 * Post test 7 for split file Schedule=Soft CL Daily. At least one variable in each 2-way table upon which measures of association are computed is a constant.
No measures of association are computed for the crosstabulation of Pretest 7 * Post test 7 for split file Schedule=Soft CL Biweekly. At least one variable in each 2-way table upon which measures of association are computed is a constant.

Case Processing Summary

Schedule		Cases			
		Valid		Missing	
		N	Percent	N	Percent
RGP permanent	Pretest 7 * Post test 7	10	100.0%	0	0.0%
Soft CL Daily	Pretest 7 * Post test 7	59	100.0%	0	0.0%
Soft CL Biweekly	Pretest 7 * Post test 7	7	100.0%	0	0.0%
Soft CL Monthly	Pretest 7 * Post test 7	56	100.0%	0	0.0%

Case Processing Summary

Schedule		Cases	
		Total	
		N	Percent
RGP permanent	Pretest 7 * Post test 7	10	100.0%
Soft CL Daily	Pretest 7 * Post test 7	59	100.0%
Soft CL Biweekly	Pretest 7 * Post test 7	7	100.0%
Soft CL Monthly	Pretest 7 * Post test 7	56	100.0%

Pretest 7 * Post test 7 Crosstabulation

Count

Schedule			Post test 7		Total
RGP permanent	Pretest 7			1	1
				9	9
	Total			10	10
Soft CL Daily	Pretest 7		2	57	59
	Total		2	57	59
Soft CL Biweekly	Pretest 7			7	7
	Total			7	7
Soft CL Monthly	Pretest 7		1	3	4
			2	50	52
	Total		3	53	56

Chi-Square Tests

Schedule		Value	df	Asymptotic Significance (2-sided)	Exact Sig. (2-sided)
RGP permanent	McNemar-Bowker Test	.	.	.[a]	
	N of Valid Cases	10			
Soft CL Daily	McNemar-Bowker Test	.	.	.[a]	
	N of Valid Cases	59			
Soft CL Biweekly	McNemar-Bowker Test	.	.	.[a]	
	N of Valid Cases	7			
Soft CL Monthly	N of Valid Cases	56			
	McNemar Test				1.000[b]

a. Computed only for a PxP table, where P must be greater than 1.

b. Binomial distribution used.

Crosstabs

Notes

Output Created		09-SEP-2022 16:35:05
Comments		
Input	Data	/Users/Jacky/Desktop/SPSS CL modality/PrePostTest_Knowledge1.sav
	Active Dataset	DataSet2
	Filter	<none>
	Weight	<none>
	Split File	Schedule
	N of Rows in Working Data File	132
Missing Value Handling	Definition of Missing	User-defined missing values are treated as missing.
	Cases Used	Statistics for each table are based on all the cases with valid data in the specified range(s) for all variables in each table.
Syntax		CROSSTABS<br>/TABLES=Pretest8 BY Posttest8<br>/FORMAT=AVALUE TABLES<br>/STATISTICS=MCNEMAR<br>/CELLS=COUNT<br>/COUNT ROUND CELL.

Notes

Resources	Processor Time	00:00:00.02
	Elapsed Time	00:00:00.00
	Dimensions Requested	2
	Cells Available	524245

Warnings

No measures of association are computed for the crosstabulation of Pretest 8 * Post test 8 for split file Schedule=RGP permanent. At least one variable in each 2-way table upon which measures of association are computed is a constant.
No measures of association are computed for the crosstabulation of Pretest 8 * Post test 8 for split file Schedule=Soft CL Daily. At least one variable in each 2-way table upon which measures of association are computed is a constant.
No measures of association are computed for the crosstabulation of Pretest 8 * Post test 8 for split file Schedule=Soft CL Biweekly. At least one variable in each 2-way table upon which measures of association are computed is a constant.

Case Processing Summary

		Cases			
		Valid		Missing	
Schedule		N	Percent	N	Percent
RGP permanent	Pretest 8 * Post test 8	10	100.0%	0	0.0%
Soft CL Daily	Pretest 8 * Post test 8	59	100.0%	0	0.0%
Soft CL Biweekly	Pretest 8 * Post test 8	7	100.0%	0	0.0%
Soft CL Monthly	Pretest 8 * Post test 8	56	100.0%	0	0.0%

Case Processing Summary

		Cases	
		Total	
Schedule		N	Percent
RGP permanent	Pretest 8 * Post test 8	10	100.0%
Soft CL Daily	Pretest 8 * Post test 8	59	100.0%
Soft CL Biweekly	Pretest 8 * Post test 8	7	100.0%
Soft CL Monthly	Pretest 8 * Post test 8	56	100.0%

Pretest 8 * Post test 8 Crosstabulation

Count

Schedule			Post test 8		Total
RGP permanent	Pretest 8			1	1
				9	9
	Total			10	10
Soft CL Daily	Pretest 8			20	20
				39	39
	Total			59	59
Soft CL Biweekly	Pretest 8			2	2
				5	5
	Total			7	7
Soft CL Monthly	Pretest 8		0	12	12
			1	43	44
	Total		1	55	56

Chi-Square Tests

Schedule		Value	df	Asymptotic Significance (2-sided)	Exact Sig. (2-sided)
RGP permanent	McNemar-Bowker Test	.	.	.[a]	
	N of Valid Cases	10			
Soft CL Daily	McNemar-Bowker Test	.	.	.[a]	
	N of Valid Cases	59			
Soft CL Biweekly	McNemar-Bowker Test	.	.	.[a]	
	N of Valid Cases	7			
Soft CL Monthly	N of Valid Cases	56			
	McNemar Test				.003[b]

a. Computed only for a PxP table, where P must be greater than 1.

b. Binomial distribution used.

Crosstabs

Notes

Output Created		09-SEP-2022 16:35:15
Comments		
Input	Data	/Users/Jacky/Desktop/SPSS CL modality/PrePostTest_Knowledge1.sav
	Active Dataset	DataSet2
	Filter	<none>
	Weight	<none>
	Split File	Schedule
	N of Rows in Working Data File	132
Missing Value Handling	Definition of Missing	User-defined missing values are treated as missing.
	Cases Used	Statistics for each table are based on all the cases with valid data in the specified range(s) for all variables in each table.
Syntax		CROSSTABS /TABLES=Pretest9 BY Posttest9 /FORMAT=AVALUE TABLES /STATISTICS=MCNEMAR /CELLS=COUNT /COUNT ROUND CELL.
Resources	Processor Time	00:00:00.02
	Elapsed Time	00:00:00.00
	Dimensions Requested	2
	Cells Available	524245

Warnings

No measures of association are computed for the crosstabulation of Pretest 9 * Post test 9 for split file Schedule=Soft CL Biweekly. At least one variable in each 2-way table upon which measures of association are computed is a constant.

Case Processing Summary

Schedule		Cases			
		Valid		Missing	
		N	Percent	N	Percent
RGP permanent	Pretest 9 * Post test 9	10	100.0%	0	0.0%
Soft CL Daily	Pretest 9 * Post test 9	59	100.0%	0	0.0%
Soft CL Biweekly	Pretest 9 * Post test 9	7	100.0%	0	0.0%
Soft CL Monthly	Pretest 9 * Post test 9	56	100.0%	0	0.0%

Case Processing Summary

Schedule		Cases	
		Total	
		N	Percent
RGP permanent	Pretest 9 * Post test 9	10	100.0%
Soft CL Daily	Pretest 9 * Post test 9	59	100.0%
Soft CL Biweekly	Pretest 9 * Post test 9	7	100.0%
Soft CL Monthly	Pretest 9 * Post test 9	56	100.0%

Pretest 9 * Post test 9 Crosstabulation

Count

Schedule			Post test 9		Total
RGP permanent	Pretest 9		1	2	3
			1	6	7
	Total		2	8	10
Soft CL Daily	Pretest 9		2	8	10
			0	49	49
	Total		2	57	59
Soft CL Biweekly	Pretest 9			7	7
	Total			7	7
Soft CL Monthly	Pretest 9		0	13	13
			4	39	43
	Total		4	52	56

Chi-Square Tests

Schedule		Value	df	Asymptotic Significance (2-sided)	Exact Sig. (2-sided)
RGP permanent	McNemar Test				1.000[a]
	N of Valid Cases	10			
Soft CL Daily	McNemar Test				.008[a]
	N of Valid Cases	59			
Soft CL Biweekly	N of Valid Cases	7			
	McNemar-Bowker Test	.	.	.[b]	
Soft CL Monthly	McNemar Test				.049[a]
	N of Valid Cases	56			

a. Binomial distribution used.

b. Computed only for a PxP table, where P must be greater than 1.

Crosstabs

Notes

Output Created		09-SEP-2022 16:35:27
Comments		
Input	Data	/Users/Jacky/Desktop/SPSS CL modality/PrePostTest_Knowledge1.sav
	Active Dataset	DataSet2
	Filter	<none>
	Weight	<none>
	Split File	Schedule
	N of Rows in Working Data File	132
Missing Value Handling	Definition of Missing	User-defined missing values are treated as missing.
	Cases Used	Statistics for each table are based on all the cases with valid data in the specified range(s) for all variables in each table.
Syntax		CROSSTABS /TABLES=Pretest10 BY Posttest10 /FORMAT=AVALUE TABLES /STATISTICS=MCNEMAR /CELLS=COUNT /COUNT ROUND CELL.

Notes

Resources	Processor Time	00:00:00.02
	Elapsed Time	00:00:00.00
	Dimensions Requested	2
	Cells Available	524245

Warnings

No measures of association are computed for the crosstabulation of Pretest 10 * Post test 10 for split file Schedule=RGP permanent. At least one variable in each 2-way table upon which measures of association are computed is a constant.
No measures of association are computed for the crosstabulation of Pretest 10 * Post test 10 for split file Schedule=Soft CL Biweekly. At least one variable in each 2-way table upon which measures of association are computed is a constant.

Case Processing Summary

		Cases			
		Valid		Missing	
Schedule		N	Percent	N	Percent
RGP permanent	Pretest 10 * Post test 10	10	100.0%	0	0.0%
Soft CL Daily	Pretest 10 * Post test 10	59	100.0%	0	0.0%
Soft CL Biweekly	Pretest 10 * Post test 10	7	100.0%	0	0.0%
Soft CL Monthly	Pretest 10 * Post test 10	56	100.0%	0	0.0%

Case Processing Summary

		Cases	
		Total	
Schedule		N	Percent
RGP permanent	Pretest 10 * Post test 10	10	100.0%
Soft CL Daily	Pretest 10 * Post test 10	59	100.0%
Soft CL Biweekly	Pretest 10 * Post test 10	7	100.0%
Soft CL Monthly	Pretest 10 * Post test 10	56	100.0%

Pretest 10 * Post test 10 Crosstabulation

Count

Schedule			Post test 10		Total
RGP permanent	Pretest 10			10	10
	Total			10	10
Soft CL Daily	Pretest 10		3	54	57
			1	1	2
	Total		4	55	59
Soft CL Biweekly	Pretest 10			6	6
				1	1
	Total			7	7
Soft CL Monthly	Pretest 10		15	37	52
			1	3	4
	Total		16	40	56

Chi-Square Tests

Schedule		Value	df	Asymptotic Significance (2-sided)	Exact Sig. (2-sided)
RGP permanent	McNemar-Bowker Test	.	.	.[a]	
	N of Valid Cases	10			
Soft CL Daily	N of Valid Cases	59			
	McNemar Test				<.001[b]
Soft CL Biweekly	McNemar-Bowker Test	.	.	.[a]	
	N of Valid Cases	7			
Soft CL Monthly	N of Valid Cases	56			
	McNemar Test				<.001[b]

a. Computed only for a PxP table, where P must be greater than 1.

b. Binomial distribution used.

Crosstabs

Notes

Output Created		09-SEP-2022 16:35:36
Comments		
Input	Data	/Users/Jacky/Desktop/SPSS CL modality/PrePostTest_Knowledge1.sav
	Active Dataset	DataSet2
	Filter	<none>
	Weight	<none>
	Split File	Schedule
	N of Rows in Working Data File	132
Missing Value Handling	Definition of Missing	User-defined missing values are treated as missing.
	Cases Used	Statistics for each table are based on all the cases with valid data in the specified range(s) for all variables in each table.
Syntax		CROSSTABS /TABLES=Pretest11 BY Posttest11 /FORMAT=AVALUE TABLES /STATISTICS=MCNEMAR /CELLS=COUNT /COUNT ROUND CELL.
Resources	Processor Time	00:00:00.02
	Elapsed Time	00:00:00.00
	Dimensions Requested	2
	Cells Available	524245

Warnings

No measures of association are computed for the crosstabulation of Pretest 11 * Posttest 11 for split file Schedule=RGP permanent. At least one variable in each 2-way table upon which measures of association are computed is a constant.
No measures of association are computed for the crosstabulation of Pretest 11 * Posttest 11 for split file Schedule=Soft CL Daily. At least one variable in each 2-way table upon which measures of association are computed is a constant.
No measures of association are computed for the crosstabulation of Pretest 11 * Posttest 11 for split file Schedule=Soft CL Biweekly. At least one variable in each 2-way table upon which measures of association are computed is a constant.

Case Processing Summary

Schedule		Cases: Valid N	Valid Percent	Missing N	Missing Percent
RGP permanent	Pretest 11 * Posttest 11	10	100.0%	0	0.0%
Soft CL Daily	Pretest 11 * Posttest 11	59	100.0%	0	0.0%
Soft CL Biweekly	Pretest 11 * Posttest 11	7	100.0%	0	0.0%
Soft CL Monthly	Pretest 11 * Posttest 11	56	100.0%	0	0.0%

Case Processing Summary

Schedule		Cases: Total N	Total Percent
RGP permanent	Pretest 11 * Posttest 11	10	100.0%
Soft CL Daily	Pretest 11 * Posttest 11	59	100.0%
Soft CL Biweekly	Pretest 11 * Posttest 11	7	100.0%
Soft CL Monthly	Pretest 11 * Posttest 11	56	100.0%

Pretest 11 * Posttest 11 Crosstabulation

Count

Schedule			Posttest 11		Total
RGP permanent	Pretest 11			10	10
	Total			10	10
Soft CL Daily	Pretest 11			59	59
	Total			59	59
Soft CL Biweekly	Pretest 11			7	7
	Total			7	7
Soft CL Monthly	Pretest 11		23	30	53
			0	3	3
	Total		23	33	56

Chi-Square Tests

Schedule		Value	df	Asymptotic Significance (2-sided)	Exact Sig. (2-sided)
RGP permanent	McNemar-Bowker Test	.	.	.[a]	
	N of Valid Cases	10			
Soft CL Daily	McNemar-Bowker Test	.	.	.[a]	
	N of Valid Cases	59			
Soft CL Biweekly	McNemar-Bowker Test	.	.	.[a]	
	N of Valid Cases	7			
Soft CL Monthly	N of Valid Cases	56			
	McNemar Test				<.001[b]

a. Computed only for a PxP table, where P must be greater than 1.

b. Binomial distribution used.

Crosstabs

Notes

Output Created		09-SEP-2022 16:35:47
Comments		
Input	Data	/Users/Jacky/Desktop/SPSS CL modality/PrePostTest_Knowledge1.sav
	Active Dataset	DataSet2
	Filter	<none>
	Weight	<none>
	Split File	Schedule
	N of Rows in Working Data File	132
Missing Value Handling	Definition of Missing	User-defined missing values are treated as missing.
	Cases Used	Statistics for each table are based on all the cases with valid data in the specified range(s) for all variables in each table.
Syntax		CROSSTABS /TABLES=Pretest12 BY Posttest12 /FORMAT=AVALUE TABLES /STATISTICS=MCNEMAR /CELLS=COUNT /COUNT ROUND CELL.

Notes

Resources	Processor Time	00:00:00.02
	Elapsed Time	00:00:00.00
	Dimensions Requested	2
	Cells Available	524245

Warnings

No measures of association are computed for the crosstabulation of Pretest 12 * Posttest 12 for split file Schedule=RGP permanent. At least one variable in each 2-way table upon which measures of association are computed is a constant.
No measures of association are computed for the crosstabulation of Pretest 12 * Posttest 12 for split file Schedule=Soft CL Biweekly. At least one variable in each 2-way table upon which measures of association are computed is a constant.

Case Processing Summary

		Cases			
		Valid		Missing	
Schedule		N	Percent	N	Percent
RGP permanent	Pretest 12 * Posttest 12	10	100.0%	0	0.0%
Soft CL Daily	Pretest 12 * Posttest 12	59	100.0%	0	0.0%
Soft CL Biweekly	Pretest 12 * Posttest 12	7	100.0%	0	0.0%
Soft CL Monthly	Pretest 12 * Posttest 12	56	100.0%	0	0.0%

Case Processing Summary

		Cases	
		Total	
Schedule		N	Percent
RGP permanent	Pretest 12 * Posttest 12	10	100.0%
Soft CL Daily	Pretest 12 * Posttest 12	59	100.0%
Soft CL Biweekly	Pretest 12 * Posttest 12	7	100.0%
Soft CL Monthly	Pretest 12 * Posttest 12	56	100.0%

Pretest 12 * Posttest 12 Crosstabulation

Count

Schedule			Posttest 12		Total
RGP permanent	Pretest 12			6	6
				4	4
	Total			10	10
Soft CL Daily	Pretest 12		2	51	53
			0	6	6
	Total		2	57	59
Soft CL Biweekly	Pretest 12			6	6
				1	1
	Total			7	7
Soft CL Monthly	Pretest 12		7	16	23
			2	31	33
	Total		9	47	56

Chi-Square Tests

Schedule		Value	df	Asymptotic Significance (2-sided)	Exact Sig. (2-sided)
RGP permanent	McNemar-Bowker Test	.	.	.[a]	
	N of Valid Cases	10			
Soft CL Daily	N of Valid Cases	59			
	McNemar Test				<.001[b]
Soft CL Biweekly	McNemar-Bowker Test	.	.	.[a]	
	N of Valid Cases	7			
Soft CL Monthly	N of Valid Cases	56			
	McNemar Test				.001[b]

a. Computed only for a PxP table, where P must be greater than 1.

b. Binomial distribution used.

Crosstabs

Notes

Output Created		09-SEP-2022 16:35:58
Comments		
Input	Data	/Users/Jacky/Desktop/SPSS CL modality/PrePostTest_Knowledge1.sav
	Active Dataset	DataSet2
	Filter	<none>
	Weight	<none>
	Split File	Schedule
	N of Rows in Working Data File	132
Missing Value Handling	Definition of Missing	User-defined missing values are treated as missing.
	Cases Used	Statistics for each table are based on all the cases with valid data in the specified range(s) for all variables in each table.
Syntax		CROSSTABS /TABLES=Pretest13 BY Posttest13 /FORMAT=AVALUE TABLES /STATISTICS=MCNEMAR /CELLS=COUNT /COUNT ROUND CELL.
Resources	Processor Time	00:00:00.02
	Elapsed Time	00:00:00.00
	Dimensions Requested	2
	Cells Available	524245

Warnings

No measures of association are computed for the crosstabulation of Pretest 13 * Posttest 13 for split file Schedule=RGP permanent. At least one variable in each 2-way table upon which measures of association are computed is a constant.

Case Processing Summary

Schedule		Cases Valid N	Valid Percent	Missing N	Missing Percent
RGP permanent	Pretest 13 * Posttest 13	10	100.0%	0	0.0%
Soft CL Daily	Pretest 13 * Posttest 13	57	96.6%	2	3.4%
Soft CL Biweekly	Pretest 13 * Posttest 13	7	100.0%	0	0.0%
Soft CL Monthly	Pretest 13 * Posttest 13	56	100.0%	0	0.0%

Case Processing Summary

Schedule		Cases Total N	Total Percent
RGP permanent	Pretest 13 * Posttest 13	10	100.0%
Soft CL Daily	Pretest 13 * Posttest 13	59	100.0%
Soft CL Biweekly	Pretest 13 * Posttest 13	7	100.0%
Soft CL Monthly	Pretest 13 * Posttest 13	56	100.0%

Pretest 13 * Posttest 13 Crosstabulation

Count

Schedule			Posttest 13		Total
RGP permanent	Pretest 13		1	9	10
	Total		1	9	10
Soft CL Daily	Pretest 13		2	43	45
			0	12	12
	Total		2	55	57
Soft CL Biweekly	Pretest 13		1	4	5
			0	2	2
	Total		1	6	7
Soft CL Monthly	Pretest 13		13	16	29
			0	27	27
	Total		13	43	56

Chi-Square Tests

Schedule		Value	df	Asymptotic Significance (2-sided)	Exact Sig. (2-sided)
RGP permanent	McNemar-Bowker Test	.	.	.[a]	
	N of Valid Cases	10			
Soft CL Daily	N of Valid Cases	57			
	McNemar Test				<.001[b]
Soft CL Biweekly	N of Valid Cases	7			
	McNemar Test				.125[b]
Soft CL Monthly	N of Valid Cases	56			
	McNemar Test				<.001[b]

a. Computed only for a PxP table, where P must be greater than 1.

b. Binomial distribution used.
